# Which lumbar spinal stenosis patients will improve with nonsurgical treatment? A secondary analysis of a randomized controlled trial

**DOI:** 10.1186/s12998-025-00620-0

**Published:** 2025-12-09

**Authors:** Eric J. Roseen, Clair N. Smith, Asifa Rahim, Conor Deal, Ryan Fischer, Natalia E. Morone, Andrew Flack, Charles Penza, Pradeep Suri, Paul E. Dougherty, Debra K. Weiner, Michael J. Schneider

**Affiliations:** 1 Section of General Internal Medicine, Department of Medicine, Boston University Chobanian and Avedisian School of Medicine and Boston Medical Center, Boston, MA USA; 2 SHRS Data Center, School of Health and Rehabilitation Sciences, University of Pittsburgh, Pittsburgh, PA USA; 3 Orlando VA Medical Center, Orlando, FL USA; 4 Clinical Learning, Evidence, and Research Center, University of Washington and Department of Rehabilitation Medicine, University of Washington, Seattle, WA USA; 5 VA Puget Sound Health Care System, Seattle, WA USA; 6 Center for Integrated Healthcare, Syracuse VA Medical Center, Syracuse, NY USA; 7 Division of Geriatric Medicine, Department of Medicine; Department of Psychiatry; Department of Anesthesiology; Clinical and Translational Sciences Institute, University of Pittsburgh, Pittsburgh, PA USA; 8 Doctor of Chiropractic Program, School of Health and Rehabilitation Sciences, University of Pittsburgh, Pittsburgh, PA USA

**Keywords:** Spinal stenosis, Chronic pain, Nonpharmacologic treatment, Group exercise, Walking, Primary care, Older adults

## Abstract

**Background:**

Lumbar spinal stenosis (LSS) can be disabling and is a leading reason for spinal surgery in older adults. While nonsurgical treatments are recommended as first-line treatment, it remains unclear which patients will benefit most.

**Purpose:**

To identify patient characteristics associated with larger improvements or larger treatment effects among adults receiving nonsurgical LSS interventions.

**Design:**

Secondary analysis of a randomized controlled trial.

**Setting:**

Outpatient research clinics.

**Subjects:**

216 older adults with symptomatic LSS.

**Methods:**

Participants, recruited from November 2013 to June 2016, were randomized to receive: (1) manual therapy with an individualized exercise program (MTE); (2) a group exercise (GE) program; or (3) medical care (MC). We evaluated the association of baseline characteristics with 2-month change in primary outcomes: symptoms and function on the Swiss Spinal Stenosis questionnaire (SSSQ); and walking capacity in meters (m) on the self-paced walking test (SPWT). Baseline characteristics included sociodemographic and clinical variables. To explore heterogeneity of treatment effects, we evaluated unadjusted stratified estimates when comparing MTE to GE/MC. Additionally, we included an interaction term in models to test for statistical interaction.

**Results:**

At baseline, participants (mean age = 72, 54% female, 23% non-white) had moderate LSS-related symptoms/impairment (mean SSSQ score = 31.3) and limited walking capacity on SPWT (mean = 451 m). The overall improvement on SSSQ was 2.5 points with larger improvements observed among younger, non-white, non-smoking participants, and those with worse baseline LSS or back-related symptoms/impairment. Overall improvement on the SPWT was 205 m with larger improvements observed among younger participants, those with higher baseline physical activity levels and participants without knee osteoarthritis. For SSSQ, the treatment effect was larger among adults aged < 70 versus older adults (MTE vs. GE/MC; mean difference [MD] = − 4.06, 95% CI = − 6.29 to − 1.83 vs. MD = − 0.47. 95% CI = − 2.63 to 1.69, respectively; *p*-for-interaction = 0.02). For walking capacity, the treatment effect was larger among adults with hip osteoarthritis compared to those without (MTE vs. GE/MC; MD = 500 m, 95% CI = 71 to 929, vs MD = 13 m, 95% CI = − 120 to 147, respectively; *p*-for-interaction = 0.007).

**Conclusions:**

In a sample receiving nonsurgical treatments for LSS, we identified patient-level characteristics associated with larger improvements and/or treatment effects. If confirmed in larger randomized controlled trials, these findings may guide clinical decision-making to enhance clinical outcomes.

**ClinicalTrials.gov identifier:**

NCT01943435.

**Supplementary Information:**

The online version contains supplementary material available at 10.1186/s12998-025-00620-0.

## Introduction

Lumbar spinal stenosis (LSS) is a highly prevalent and disabling cause of low back and leg pain in older adults, typically characterized by worsening symptoms with lumbar extension and improved symptoms with lumbar flexion [1–4]. Adults with LSS often do not meet guidelines for physical activity due to persistent back and leg symptoms that limit walking and other daily activities, putting them at risk of functional decline and early mortality [5–7]. Furthermore, LSS is the most common reason for elective surgery in adults over the age of 65, contributing to high healthcare costs for those afflicted [8, 9]. Patients typically experience relief immediately following surgery, but often regress to their previous pain levels in long-term follow-up [10–12]. Surgery carries inherent risks and may not be suitable for all patients, particularly those who have not exhausted nonsurgical treatment options [13]. Thus, a stepped approach to care beginning with nonsurgical treatment is recommended in clinical practice guidelines, with several nonsurgical treatments being considered safe and effective for LSS [14, 15]. Determining who could benefit most from nonsurgical treatment would help guide care, avoid unnecessary surgery, and reduce healthcare costs.

Predictors of improvement (i.e., prognostic factors) have been described for older adults with LSS undergoing surgery, e.g., higher walking capacity and the absence of psychological factors before surgery are associated with better post-surgical outcome measures such as self-reported symptom severity or walking capacity [16–18]. However, few studies have evaluated predictors of improvement in nonsurgical cohorts. Prior studies have noted that walking capacity [16, 19] and psychosocial factors [20, 21] may also predict outcomes in nonsurgical LSS populations. Several patient characteristics that contribute to LSS development may be important prognostic factors, e.g., older age, obesity, smoking [22, 23]. However, it remains unclear if these factors predict LSS outcomes independent of treatment. Our study will evaluate whether there are predictors of improvement among patients receiving non surgical approaches for LSS.

Heterogeneity of treatment effects (i.e., treatment effect modification) by baseline characteristics has the potential to guide treatment selection [24–26]. Indeed, identifying tangible patient characteristics that delineate subgroups of patients that are expected to have larger or smaller treatment effects is a longstanding priority for low back pain research agendas [27, 28]. However, these analyses are challenging as they are ideally performed using data from a large randomized controlled trial with measurement of potential effect modifiers prior to treatment allocation [29]. Given that LSS is a unique patient population with different clinical outcomes, such as stenosis-related symptoms or function and walking capacity, additional studies are needed to identify treatment effect modifiers of LSS treatments. To our knowledge, no prior studies have evaluated effect modification when comparing nonpharmacologic treatments. Addressing this gap in current literature is important in identifying which patients are most likely to benefit from a clinician-delivered treatment such as manual therapy and individualized exercise compared to less-intensive approaches such as general exercise programs or usual medical care. Thus, our study will also evaluate whether there are treatment effect modifiers of a manual therapy and exercise intervention when compared to other nonsurgical approaches for LSS.

A randomized controlled trial by Schneider et al., reported that a combination of manual therapy and an individualized exercise program provides greater short-term improvement in stenosis-related symptoms and physical function and walking capacity than medical care or a group exercise program [30, 31]. We performed secondary analyses of the data from this trial to explore two additional important clinical questions: First, what pre-treatment patient characteristics may be important predictors of improvement in a nonsurgical LSS patient population? Second, which pre-treatment patient characteristics potentially explain heterogeneity of treatment effects when comparing manual therapy and individualized exercise to medical care or a group exercise program?

## Methods

### Design

This is a secondary analysis of a three-arm RCT with details on study design and main outcomes reported elsewhere [30, 31]. A total of 259 participants with symptomatic LSS were randomized to one of the following three intervention groups: (1) manual therapy and individualized exercise; (2) group exercise; and (3) medical care. For analyses in this manuscript evaluating treatment effects, we collapsed group exercise and medical care groups into a single comparison group, i.e., to compare manual therapy and individualized exercise to other nonsurgical treatment options. All interventions were delivered over a 6-week period with the current analyses focusing on short-term follow-up, i.e., primary outcomes at the 2-month endpoint. We included 216 (83%) participants with complete 2-month outcome data in these analyses [31]. Participant recruitment and follow-up occurred from November 2013 to June 2016.

### Participants

Adults over the age of 60 years with LSS were eligible if they had evidence of central and/or lateral canal stenosis on magnetic resonance imaging (MRI) or computed tomography (CT) and clinical symptoms associated with LSS (neurogenic claudication; less symptoms with flexion).

### Interventions

#### Manual therapy and individualized exercise (MTE)

This intervention group involved twice-weekly sessions for 6 weeks (12 total sessions) with participants assigned to either a chiropractor or physical therapist. These clinicians delivered a standardized protocol including a warm-up on an exercise bike, manual therapy techniques (mobilization of spine, hips, muscles, nerves and other surrounding soft tissues), and personalized stretching and strengthening exercises based on individual needs. This intervention was aimed at managing symptoms by improving mobility of the lower back and hip joints, nerves and muscles [31–33].

#### Group exercise (GE)

This intervention group participated in twice-weekly supervised exercise classes for older adults for 6 weeks (12 total sessions). The group exercise classes were 45 min in length and led by certified exercise instructors. Participants were allowed to self-select between easy and medium intensity exercise classes. This intervention was aimed at improving overall physical fitness with general exercises in a group setting (non-individualized).

#### Medical care (MC)

This intervention group received a 6-week pain management program delivered by a physiatrist. Participants initially received a combination of first-line prescribed oral medications (non-narcotic analgesics, anticonvulsants, or antidepressants) tailored to their specific needs. The physiatrist provided participants with general recommendations for gentle stretching and advice to stay active, to complement the medication management plan. If oral medications provided inadequate pain relief, participants were offered epidural steroid injections as a second-line treatment option. The decision to receive injections (which occurred in 20% of participants) also involved shared decision-making, considering factors like patient preference and neurogenic claudication severity. This intervention aimed to improve pain management through individualized medication, basic lifestyle modifications, and optional injections.

### Measures

#### Primary outcomes

Consistent with the original trial, our pre-specified primary outcomes were symptom severity and function on the Swiss Spinal Stenosis Questionnaire (SSSQ) and walking capacity on the self-paced walking test (SPWT) [30, 31]. We used the SSSQ total score, which was calculated by combining the symptom domain sub-score (7-items, scores range: 7–35) with the function domain sub-score (5-items, scores range: 5–20) [30, 31]. Thus, SSSQ total scores can range from 12 to 55 with higher scores indicating worse symptom severity and more impaired function [34–37]. Walking capacity on the SPWT was the distance in meters (m) walked before stopping due to symptoms with a maximum duration of 30 min [38, 39].

#### Baseline characteristics

To identify relevant baseline characteristics, we performed a literature review in a single database (i.e., PubMed) to identify studies that evaluated: (1) potential predictors of LSS outcomes using validated questionnaires (e.g., SSSQ) or an objective measure of walking capacity; and/or (2) treatment effect modifiers in LSS intervention trials that measured these outcomes. Additional details and findings from this review are shown in Supplemental file 1 (Appendix Tables 1–4). Given a limited number of prior studies, novel baseline characteristics (i.e., those not evaluated in previous studies) were included if there was consensus from the research team that the characteristic could theoretically influence prognosis or treatment effects. The team includes researchers and clinicians who have previously applied relevant theoretical frameworks such as the WHO’s International Classification of Functioning, Disability and Health (ICF) framework and the biopsychosocial model in older adults with pain conditions [18, 40]. To form clinically-meaningful subgroups we used established clinical cut points when available, e.g., to define obesity and severe back-related disability on the Oswestry Disability Index [41]. If established cut points were not available for continuous measures (e.g., age, baseline physical activity levels), we selected a cut point value near the median value to allow for more stable strata-specific estimates.

### Sociodemographic characteristics

Sociodemographic characteristics included age (< 70, ≥ 70 years), sex (male, female), race (white, non-white), marital status (married, not married), and household income (< $40,000/year, ≥ $40,000/year).

### Clinical characteristics

General health characteristics included obesity (≤ 30, > 30 Body Mass Index) and smoking status (never, prior/current smoker). The number of comorbid health conditions (≤ 4, > 4) was identified from the Modified Comorbidity Disease Index. The presence of co-occurring hip or knee osteoarthritis (yes, no) were evaluated on physical examination using clinical criteria from the American College of Rheumatology (ACR) [42, 43]. Participants were administered an ankle-brachial index test, a measure used to indicate the presence of peripheral artery disease that may cause leg pain, with lower values indicating more narrowing or blockage of lower extremity arteries (< 1, ≥ 1 indicating those with or without vascular claudication). We also included clinically important thresholds for baseline gait speed (< 1 m/s, ≥ 1 m/s), meters walked in a self-paced walking test (< 280 m, ≥ 28 0 m), and amount of physical activity (< 165 min/day, ≥ 165 min/day) measured by accelerometer data averaged over one week (SenseWear; BodyMedia Inc).

Back-related variables included back and leg symptom duration (≤ 6, > 6 month), and back/leg pain intensity (≤ 6, > 6) on the 0–10 numerical rating scale. Imaging interpretation from prior MRI or CT reports were reviewed to identify the presence (yes/no) of central canal stenosis, lateral recess stenosis, and intervertebral foramen stenosis [31]. From these, we characterized participants as having lateral canal stenosis only (lateral recess or intervetebral foramen), central canal stenosis only, or both central and lateral canal stenosis. We used the Oswestry Disability Index to define mild to moderate back-related disability versus severe disability (≤ 40 and > 40, respectively) [41]. The median total SSSQ score (> 31) was also used. We identified individuals with at least mild depressive symptoms on the short-form version of the PROMIS depression scale (t-score ≥ 55) [44]. We identified individuals with higher levels of kinesiophobia using the median value of ≥ 26 for the 11-item shortform of the Tampa Kinesiophobia Scale; which has scores from 11 to 44 with higher scores indicating worse kinesophobia [45]. Participants were asked about their expectations of each of the interventions prior to randomization using a 5-item pain-specific expectations questionnaire; potential scores range from 9 to 54 with higher scores indicating more favorable expectations [46]. We used responses specific to MTE, and identified those who were above the median score of 43, with scores above this value indicating favorable expectations for MTE.

### Data analysis

Baseline characteristics across the three treatment groups were summarized using means (SD) for continuous variables and frequencies (%) for categorical variables.

We took a descriptive approach to evaluating predictors of improvement and heterogeneity of treatment effects [47]. This approach emphasizes stratum-specific changes or effect estimates, and not just the results of statistical tests, i.e., we focus primarily on direction and magnitude of change or effect estimate and 95% confidence intervals (CIs), although 95% CIs are based on a *p*-value of 0.05 [47]. Thus, analyses were considered to be exploratory and hypothesis generating. For predictors this meant evaluating the magnitude of the within- and between- stratum changes in outcomes (e.g., changes in outcomes among all male or female participants). For treatment effect modification, this meant evaluating the magnitude of treatment effects within each stratum of baseline characteristics (e.g., treatment effect when comparing MTE to GE/MC among all male or female participants).

We evaluated predictors of improvement using our primary outcomes, changes in stenosis-related symptoms and physical function on the SSSQ and walking capacity on the SPWT. We calculated the mean change scores for each measure from baseline to the 2-month endpoint. We report the overall change score (i.e., among all participants) and change scores for each stratum of the baseline characteristics (i.e., among subgroups). Additionally, we calculated the between strata differences and their 95% confidence intervals to assess associations between baseline characteristics and changes in outcomes at 2 months. Forest plots were used to illustrate stratum-specific estimates of change with their 95% confidence intervals stratified by baseline characteristics.

We evaluated treatment effect modification using the specific contrast of manual therapy and individual exercise compared to all other participants, i.e., combining participants who received group exercise or medical care into one comparison group. To explore heterogeneity, we calculated the overall 2-month treatment effect (i.e., all participants) and treatment effects in each stratum of the baseline characteristics (i.e., within subgroups). Forest plots were used to illustrate stratum-specific 2-month treatment effect estimates with their 95% confidence intervals stratified by baseline characteristics. Statistical interaction was assessed with linear regression models that included effects for the potential moderator, the treatment group, and an interaction term (moderator*treatment group). Statistically significant interaction terms (*p* < 0.05) were considered evidence of potential effect modification while a *p*-value of 0.05 to 0.20 indicated exploratory evidence for potential effect modification [25, 26].

To aid interpretation of the magnitude of changes, we identified a range of values indicating a minimal clinically important difference (MCID) for each scale [48]. MCIDs for improvements in stenosis-related symptoms and physical function on the SSSQ calculated previously from our sample using multiple approaches ranged from 4.2 to 5.5 points [36]. MCIDs for improvements in walking capacity on the SPWT from multiple prior studies ranged from 319 to 376 m [36, 49, 50]. We identified strata (i.e., subgroups) where the overall or within-group average improvement (i.e., changes within the MTE, GE, or MC groups) met some or all of the pre-specified thresholds, i.e. scores within or above the MCID range, respectively.

## Results

### Sample characteristics

Among 216 participants with 2-month outcome data, 75 received manual therapy and individualized exercise, 65 received group exercise, and 76 received medical care. Participant characteristics (mean age = 72, 54% female, 23% non-white), are presented in Table 1. Participants reported moderate stenosis symptoms and functional impairment (mean SSSQ score = 31.3) and limited walking capacity (mean = 451 m) at baseline.

**Table 1:** Baseline characteristics of 216 participants

Characteristics	Medical care (*n* = 76)	Group exercise (*n* = 65)	MTE (*n* = 75)	Total (*n* = 216)
Age, mean (SD)	71.8 (6.9)	73.1 (7.9)	72.4 (8.1)	72.4 (7.6)
Age ≥ 70	37 (49)	39 (60)	40 (53)	116 (54)
Female sex	41 (54)	30 (46)	45 (60)	116 (54)
Non-white race	18/75 (24)	15 (23)	17 (23)	50/215 (23)
Married	37 (49)	34 (52)	41 (55)	112 (52)
Household income < 40,000/y	37/73 (49)	31/62 (48)	38/73 (51)	102/208 (47)
BMI > 30	42 (55)	26 (40)	33 (44)	115 (53)
Prior/current smoker	43/73 (57)	37/64 (57)	40 (53)	120/212 (56)
Duration of back symptoms, > 6 mo	70 (92)	55 (85)	69 (92)	194 (90)
Duration of leg symptoms, > 6 mo	57 (57)	44 (68)	57 (76)	158 (73)
Diagnostic imaging results				
Central canal stenosis only	6/75 (8)	5/61 (2)	6/72 (8)	17/208 (8)
Lateral canal stenosis only^a^	38/75 (51)	20/61 (33)	30/72 (42)	88/208 (42)
Central and lateral canal stenosis	31/75 (41)	36/61 (59)	36/72 (50)	103/208 (50)
Hip osteoarthritis	12 (16)	10 (15)	13 (17)	35 (16)
Knee osteoarthritis	28 (37)	16 (25)	24 (32)	68 (31)
> 4 comorbidities	40 (53)	22 (34)	37 (49)	99 (46)
Ankle-brachial index, < 1	23 (30)	25 (38)	29 (39)	77 (36)
Swiss Spinal Stenosis total score, > 31	37 (49)	35 (54)	35 (47)	107 (50)
Self-paced walking test, < 280 m	42 (55)	34 (52)	35 (47)	111 (51)
Oswestry Disability Index, > 40	32 (42)	28 (43)	28 (37)	88 (41)
Leg pain intensity, > 6	32 (42)	26 (40)	27 (36)	85 (39)
Back pain intensity, > 6	48 (63)	30 (46)	44 (59)	122 (56)
Gait speed, < 1 m/s	50 (66)	38 (58)	47 (63)	135 (63)
Physical activity, < 165 min/d	43/74 (57)	38/62 (58)	40/74 (53)	121/210 (56)
PROMIS depression, ≥ 55	15 (20)	15 (23)	12/73 (16)	42/214 (20)
Tampa Scale of Kinesiophobia, ≥ 26	44 (58)	33 (51)	36 (48)	113 (52)
High expectations for MTE, > 42	47 (62)	37 (57)	46 (61)	130 (60)

^a^Lateral canal stenosis including lateral recess or intervertebral foramen stenosis

### Predictors of change

**Fig. 1:**
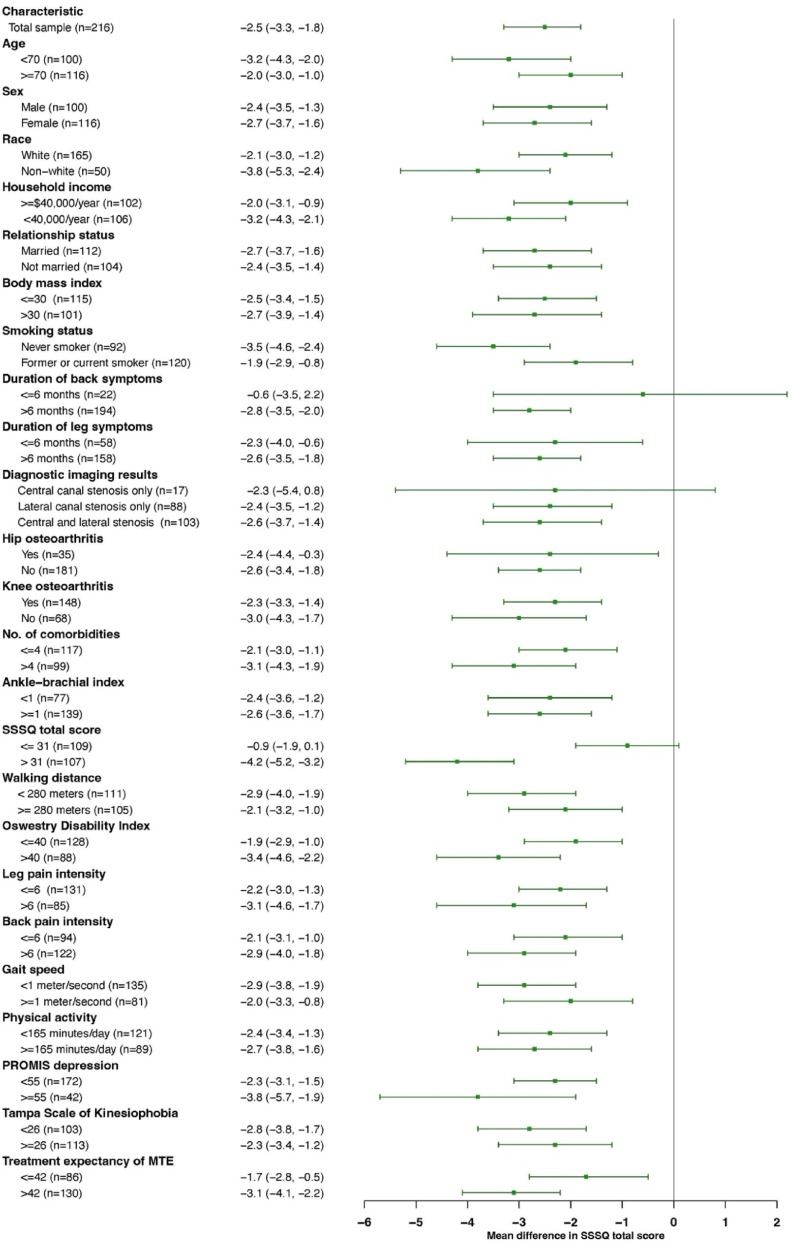
Forest plot illustrating 2-month changes in symptom severity and function on the Swiss Spinal Stenosis questionnaire stratified by baseline characteristics

Overall and stratified changes in stenosis-related symptoms and physical function on the SSSQ are shown in Fig. 1 and Appendix Table 5. The overall improvement on SSSQ was 2.5 points with larger improvements observed among younger adults (age < 70 vs. ≥ 70 years, − 3.2 ± 5.7 vs. − 2.0 ± 5.6, MD = 1.1, 95% CI = − 0.37 to 2.70), those self-reporting non-white race (vs. white race, − 3.8 ± 5.1 vs. − 2.1 ± 5.7, MD = − 1.73, 95% CI = − 3.51 to 0.05), and those with higher symptom severity and physical function burden at baseline on the SSSQ (> 31 vs. ≤ 31, − 4.2 ± 5.4 vs. − 0.9 ± 5.3, MD = − 3.27, 95% CI = − 4.72 to − 1.82) or the Oswestry Disability Index (≥ 40 vs. ≤ 40, − 3.4 ± 5.7 vs. − 1.9 ± 5.5, MD = − 1.48, 95% CI = − 3.00 to 0.05). Individuals who had higher expectations for MTE prior to randomization reported larger improvements on SSSQ compared to those with lower expectations using a pain-specific expectations questionnaire (> 42 vs. ≤ 42, − 3.1 ± 5.7 vs. − 1.7 ± 5.5, MD = − 1.49 (− 3.02 to 0.05). Smokers had smaller SSSQ improvements (vs. never-smokers, − 1.9 ± 5.8 vs. − 3.5 ± 5.3, MD = 1.60, 95% CI = 0.07 to 3.14). Only one stratum-specific 2-month change was within the MCID range for SSSQ of 4.2 to 5.5 points (i.e., baseline SSSQ score > 31, − 4.2 ± 5.4), and no values were above this range.

**Fig. 2:**
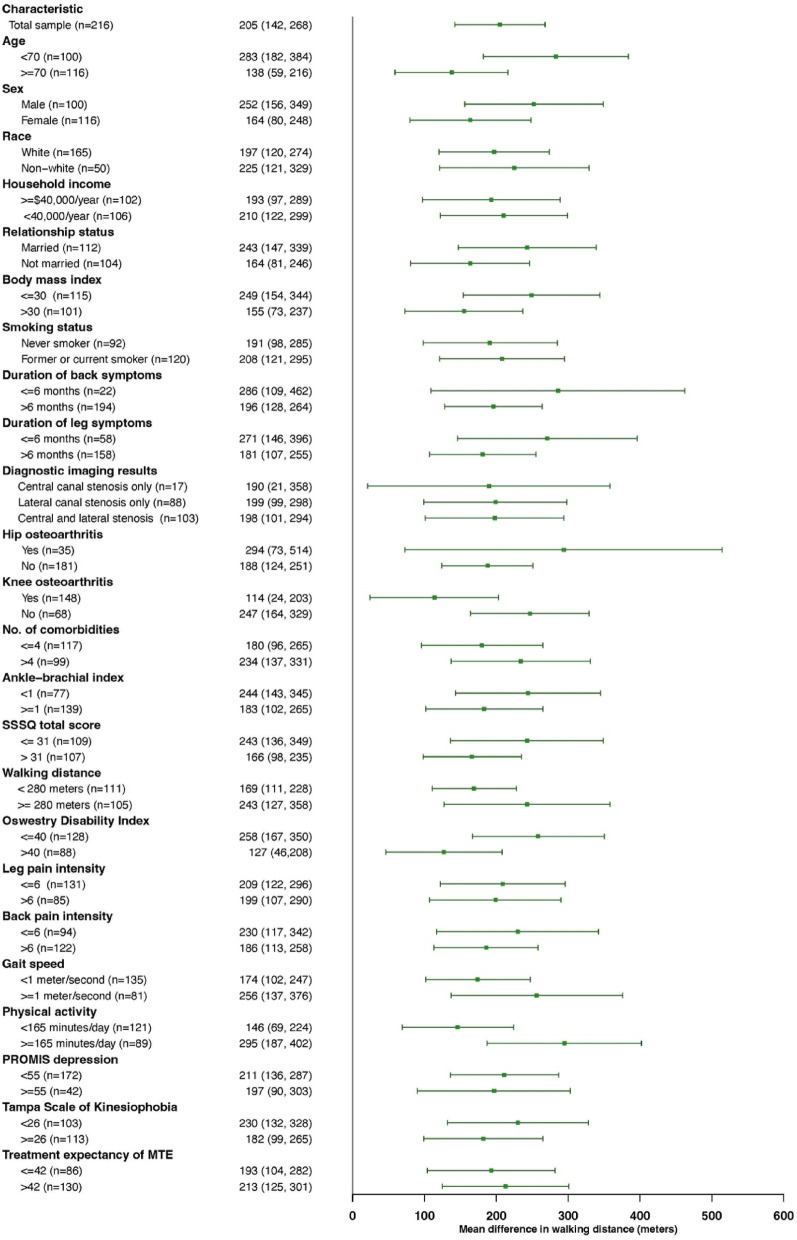
Forest plot illustrating 2-month changes in walking distance on self-paced walking test stratified by baseline characteristics

Overall and stratified changes in walking capacity on SPWT are shown in Fig. 2 and Appendix Table 6. The overall improvement on walking capacity was 205 m with larger improvements observed among younger adults (age < 70 vs. ≥ 70 years, 283 ± 511 vs. 138 ± 427, MD = 146 m, 95% CI = 20 to 271), those without knee osteoarthritis (no knee osteoarthritis vs. osteoarthritis, 247 ± 508 vs. 114 ± 371, MD = 133 m, 95% CI = 12 to 254), and individuals with higher baseline physical activity levels (≥ 165 min/day vs. < 165 min/day, 295 ± 512 vs. 146 ± 431, MD = 148 m, 95% CI = 20 to 277). Improvements in walking capacity were smaller among those with higher back-related disability on the Oswestry Disability Index (≥ 40 vs. ≤ 40, 127 ± 381 vs. 258 ± 521, MD = − 131 m, 95% CI = − 252 to − 11). No stratum-specific 2-month changes were within or above the MCID range for the self-paced walking test (i.e., 319 to 376 m).

### Potential treatment effect modifiers

**Fig. 3:**
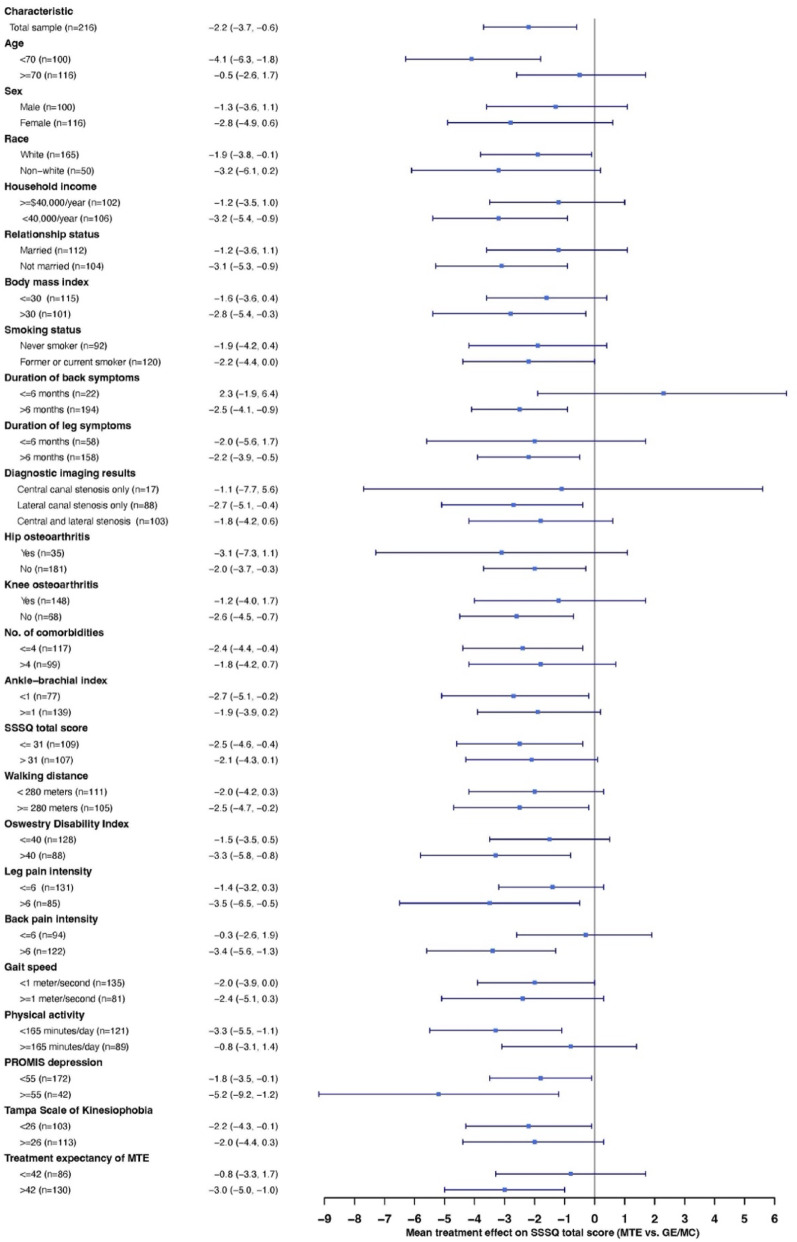
Forest plot illustrating 2-month treatment effects of manual therapy and exercise (MTE) compared to group exercise (GE) and medical care (MC) for change in symptom severity and function on the Swiss Spinal Stenosis questionnaire stratified by baseline characteristics

Overall and stratified treatment effects on SSSQ, when comparing MTE to GE/MC, are shown in Fig. 3 and Appendix Table 7. The treatment effect on SSSQ, when comparing MTE to GE/MC, was larger among adults aged < 70 versus older adults (MD = − 4.06, 95% CI = − 6.29 to − 1.83, vs. MD = − 0.47. 95% CI = − 2.63 to 1.69, respectively; *p*-for-interaction = 0.02. As shown in Appendix Table 7, four additional characteristics were under the exploratory threshold for statistical interaction: larger effect estimates were observed among individuals with longer duration of back pain symptoms (*p*-for-interaction = 0.09), individuals with at least mild depression (*p*-for-interaction = 0.10), lower baseline physical activity levels (*p*-for-interaction = 0.12), and higher expectations that MTE would be helpful (*p*-for-interaction = 0.17). As shown in Appendix Table 7, stratum-specific 2-month changes within the MTE group fell within the MCID range for SSSQ (4.2-to-5.5-point improvement) were participants reporting female sex, not married, income < $40,000/year, BMI > 30, back symptoms > 6 months, lateral canal stenosis only on imaging, hip osteoarthritis on ACR criteria, > 4 comorbidities, lower baseline walking capacity, and lower baseline physical activity levels. Larger within-group MTE changes (i.e., > 5.5 points) were observed among adults aged < 70 years (− 5.8 ± 5.7), non-white adults (− 5.9 ± 4.9), those with baseline SSSQ score above 31 (− 5.6 ± 6.1), those with baseline leg pain scores rated as > 6 on the 0–10 numerical rating scale (− 5.5 ± 7.1), and those with at least mild depressive symptoms (− 7.5 ± 6.1). None of the stratum-specific 2-month within-group changes for GE or MC were within or above the MCID range for SSSQ.

**Fig. 4:**
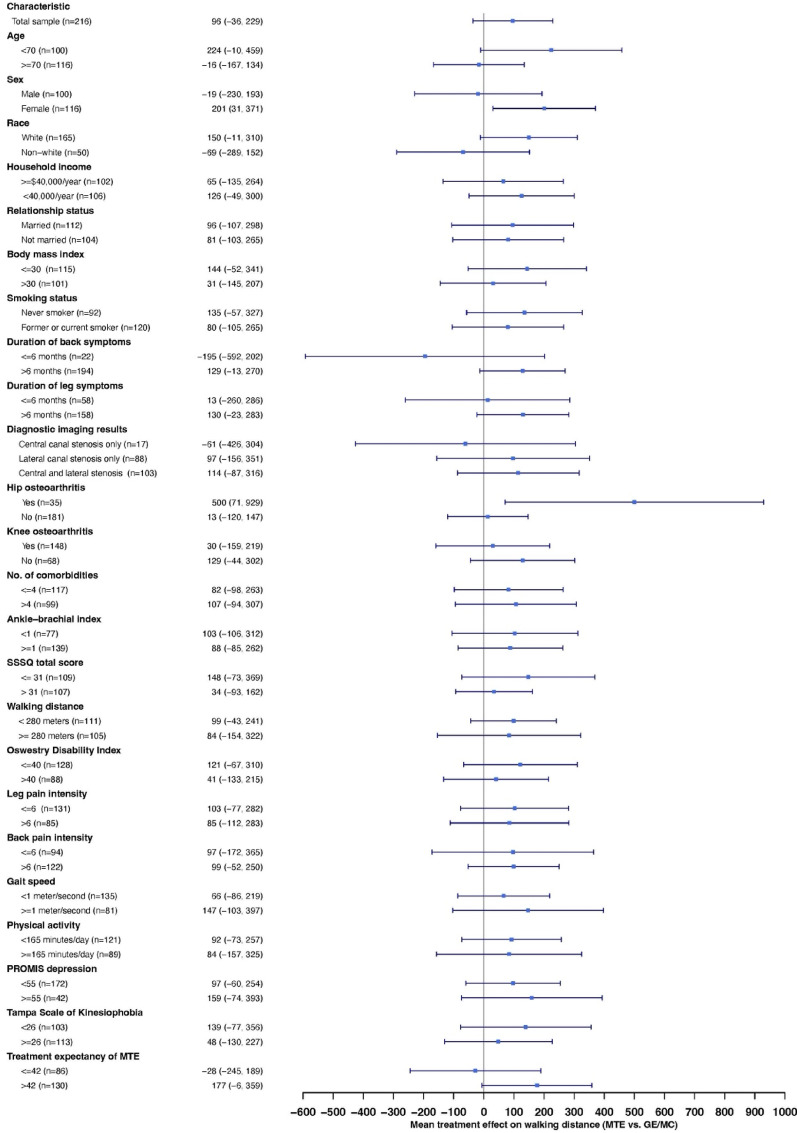
Forest plot illustrating 2-month treatment effects of manual therapy and exercise (MTE) compared to group exercise (GE) and medical care (MC) for change in walking distance on self-paced walking test stratified by baseline characteristics

Overall and stratified treatment effects on walking capacity, when comparing MTE to GE/MC, are shown in Fig. 4 and Appendix Table 8. For walking capacity, the treatment effect was larger among adults with hip osteoarthritis compared to those without (MD = 500, 95% CI = 71 to 929, vs. MD = 13, 95% CI = − 120 to 147, respectively; *p*-for-interaction = 0.01). As shown in Appendix Table 8, five additional characteristics were under the exploratory threshold for statistical interaction; larger effect estimates were observed among participants reporting younger age (< 70 years, *p*-for-interaction = 0.07), female sex (*p*-for-interaction = 0.11), white race participants (*p*-for-interaction = 0.17), a longer duration of back pain symptoms (*p*-for-interaction = 0.17), and those with higher expectations that MTE would be helpful (*p*-for-interaction = 0.14). As shown in Appendix Table 8, several stratum-specific 2-month changes within the MTE group were within the MCID range for the self-paced walking test of 319 to 376 m, including participants who at baseline had a BMI ≤ 30, no knee osteoarthritis using ACR criteria, SSSQ of 31 or less, ODI of 40 or less, gate speed ≥ 1 m/s; and measured physical activity of < 165 min per day. Larger within-group MTE changes (i.e., > 376 m) were observed for adults aged < 70 years (429 ± 616 m) and those with hip osteoarthritis using the ACR criteria (608 ± 693 m). While none of the stratum-specific 2-month within-group changes for GE or MC were above the MCID range for SPWT, some characteristics were within the range for GE (male sex, non-white race, a duration of back symptoms of 6 months or less) and MC (duration of back symptoms of 6 months or less).

## Discussion

Among older adults with lumbar spinal stenosis receiving nonsurgical treatments, several baseline characteristics were associated with 2-month changes in stenosis-related symptoms, function, and walking capacity. Greater improvements in stenosis related symptoms and function were identified among younger, non-white, and non-smoking participants, and those with worse back-related disability on the Oswestry Disability Index or higher symptom and function burden on the SSSQ. Improvements in walking capacity were also larger among younger participants, participants without knee osteoarthritis and those with higher baseline physical activity levels. Modest improvements with MTE when compared to the other interventions were relatively consistent across subgroups. However, treatment effects on SSSQ and walking capacity were potentially modified by age group and hip osteoarthritis, respectively. In other words, a large effect of MTE on SSSQ, compared to the other intervention groups, was observed among adults under the age of 70; and no treatment effect on stenosis-related symptoms and function was observed for adults over the age of 70. Similarly, the effect of MTE on walking capacity when compared to the other intervention groups was large among adults with hip osteoarthritis identified on physical exam using the ACR clinical criteria, and no treatment effect was observed on walking capacity for adults without hip osteoarthritis.

While a large number of studies have looked at prognostic factors in surgical populations [16], relatively few have evaluated predictors of stenosis-specific self-report outcome measures such as the SSSQ or objective measures of walking capacity. Findings from studies with these outcomes in populations receiving surgical interventions or procedures (e.g., spinal injections [51]) are mixed but generally support our findings of better outcomes with younger age, longer duration of pain, and more severe symptoms and worse function at baseline [18, 51–56]. While prior observational studies of pain conditions have identified Black race or Hispanic ethnicity as being associated with worse outcomes and less treatment, [57] we found non-white participants had better SSSQ outcomes compared to white participants. While we did not have information to fully explore this association, we suspect barriers to treatment observed in real world settings may be addressed, at least in part, in the context of a clinical trial where everyone is offered treatment. Our findings are also similar to two prior studies of prognostic factors in populations receiving nonpharmacologic treatments, where younger individuals and those with fewer comorbid conditions had better outcomes [58, 59]. We found that depressive symptoms were associated with changes in SSSQ total score but not walking capacity, which is consistent with prior reviews [20, 21]. However, we found that individuals with depressive symptoms had a better prognosis (i.e., larger short-term improvements in SSSQ) which is in contrast with prior studies that have found higher baseline depressive symptoms are associated with higher rates of persistent pain in surgical populations [20, 21]. Prior qualitative studies suggest the physical and psychosocial impacts of neurogenic claudication negatively impact outcomes of adults with LSS receiving nonpharmacologic treatments [37].

We are unaware of other studies of potential treatment effect modifiers for nonpharmacologic treatments for LSS. Thus, our findings should be considered exploratory and hypothesis generating. For example, our findings raise the question of whether the MTE intervention can improve objective walking capacity more than other nonsurgical treatments among individuals with LSS and comorbid hip osteoarthritis. While this finding should be interpreted with caution, particularly given the relatively small number of adults with hip osteoarthritis, it is worth further study. It is important to recognize that hip osteoarthritis itself can mimic symptoms of neurogenic claudication and that hip osteoarthritis and LSS are commonly comorbid [60, 61]. Participants in the original trial were ascribed a diagnosis of hip osteoarthritis based upon physical exam using ACR clinical criteria and X-rays were not performed [43]. It is possible, therefore, that diminished hip range of motion was caused as much by periarticular soft tissue restrictions as by pathology within the joint (i.e., degenerative disease). And, since manual therapy to the hips and associated soft tissue was part of the MTE intervention, improved walking capacity may have been related to improved hip mobility. In contrast, we did not see similar larger improvements with MTE among participants with knee osteoarthritis, perhaps because our protocol did not involve treatment of the knee. Older adults often have more than one pain condition [40], and may benefit more from treatment that is directed to all body regions that contribute to important outcomes such as walking capacity [62].

Our study had several limitations. First, we had a relatively small sample size, particularly for treatment effect modification analyses where sample size is ideally > 500 participants [29]. Second, we took a descriptive epidemiologic approach which has inherent strengths and weaknesses [47]. This approach involved stratifying changes in outcomes, and treatment effects, by baseline characteristics to identify larger than average changes or treatment effects. We presented the unadjusted values of association that have predictive value but may not have a causal interpretation. Inclusion of baseline characteristics in each analysis was guided by available theory and clinical experience of the research team. However, stratifying by many covariates runs the risk of identifying spurious associations [24, 47]. Our approach also did not allow us to stratify across multiple baseline characteristics simultaneously, which may add prognostic value. Since further stratification would result in an unmanageable number of strata, predictive models may be used to generate a risk score and to estimate changes or treatment effects within strata of that risk score [24]. The use of two samples and cross-validation is recommended when taking this approach [24, 63].

Despite the above limitations, our study also had several strengths. Our study is the first to evaluate whether there are effect modifiers of a manual therapy and exercise intervention compared to other nonsurgical approaches for LSS. By evaluating single factors, our findings should be feasible to replicate and, if they are replicated, relatively simple to implement in clinical practice. Additional studies could be combined with ours through meta-analysis to provide estimates with narrower confidence intervals. This is in contrast with prior studies that have emphasized statistical testing to indicate meaningful subgroups. Second, we leveraged MCIDs to aid interpretation of the magnitude of stratum-specific effects. For walking capacity, several prior studies have estimated MCID for the self-paced walking test as an increase in 319 to 376 m [36, 49, 50]. An increase of 400 m may allow older adults significant independence, as walking distances of 200 to 500 m are often considered walkable for older adults, allowing individuals to navigate parks, stores, and other community resources [64, 65]. For SSSQ we used and MCID range from 4.2 to 5.5 points, which is based on prior analyses of our sample [36]. However, it is important to note that SSSQ improvements > 4 points might be considered large, as prior studies have estimated MCIDs to be lower (e.g., approximately 1–2 points when combining symptom and function domains) [66, 67]. However, it is also possible that prior studies enrolled participants with more advanced disease and a worse prognosis [66, 67].

Our study represents an important first step in understanding subgroups of patients with LSS that are more likely to benefit from manual therapy and individualized exercise when compared to other nonsurgical approaches. We anticipate that our findings can inform hypotheses that can be pre-specified and tested in a future large trial. Furthermore, the use of existing frameworks, such as the Instrument for assessing the Credibility of Effect Modification Analyses (ICEMAN), can improve the credibility of effect modification analyses [68]. While our analyses may have met some of the relevant ICEMAN criteria (use of clinically important thresholds to define subgroups; statistical interaction testing), others were not met. For example, we did not pre-specify the hypothesized direction of effects in each subgroup and could not compare our subgroup treatment effects to prior studies. Consistent with our exploratory approach we evaluated many potentially important subgroups, rather than testing a small set of suspected treatment effect modifiers. Nonetheless, we acknowledge that meeting all ICEMAN criteria is important for establishing credible subgroups that can be widely implemented in usual medical care to tailor care and improve patient outcomes.

## Conclusions

Among older adults with lumbar spinal stenosis receiving nonsurgical treatments, improvements in stenosis-related symptoms and function were larger among younger, non-white, and non-smoking participants, and those with higher symptom and function burden on the SSSQ or the Oswestry Disability Index at baseline. Improvements on walking capacity were also larger among younger participants, participants without knee osteoarthritis and those with higher baseline physical activity levels. However, relatively few baseline characteristics defined subgroups of participants who had larger improvements with manual therapy and individualized exercise when compared to group exercise or usual medical care groups. Larger trials are needed to test our findings and identify additional characteristics that may guide clinical decision making for providing nonsurgical treatment options to patients with LSS.

## Supplementary Information

Below is the link to the electronic supplementary material.

Supplementary Material 1

## Data Availability

No datasets were generated or analysed during the current study.
