# Supplementary material for: Which lumbar spinal stenosis patients will improve with nonsurgical treatment? A secondary analysis of a randomized controlled trial
Source: Chiropr Man Therap. 2025 Dec 9;33:57. doi: 10.1186/s12998-025-00620-0 (PMC12690792; doi:10.1186/s12998-025-00620-0)
Supplement: Supplementary file 1 — Supplementary Material 1 [file 12998_2025_620_MOESM1_ESM.docx]

**Supplementary materials**

**Literature review** (Supplemental file 1):

**Appendix Table 1:** Search strategies

**Appendix Table 2.** Overview of studies evaluating predictors and/or effect modifiers of lumbar spinal stenosis (LSS) symptoms, function and walking capacity

**Appendix Table 3.** Predictors of lumbar spinal stenosis (LSS) symptoms, function and walking capacity

**Appendix Table 4.** Potential treatment effect modifiers of of lumbar spinal stenosis (LSS) symptoms, function and walking capacity

**Additional tables:**

**Appendix Table 5.** Predictors of 2-month change in lumbar spinal stenosis symptom severity and function on the total score of the Swiss Spinal Stenosis (SSS)

**Appendix Table 6.** Predictors of 2-month change in walking distance on self-paced walking test

**Appendix Table 7.** Treatment effect modification of Manual Therapy and Exercise compared to Group Exercise (GE) and Medical Care (MC) for change in disability on the Swiss Spinal Stenosis symptom severity scale at 2 months.

**Appendix Table 8.** Treatment effect modification of Manual Therapy and Exercise compared to Group Exercise (GE) and Medical Care (MC) for change in walking distance on self-paced walking test at 2 months.

**Literature review** (Supplemental file 1)

**APPROACH**

We conducted a literature review to identify prior studies of baseline characteristics that either predict improvement in clinical outcomes or identify treatment effect modifiers among adults with lumbar spinal stenosis. We included articles that enrolled patients with lumbar spinal stenosis but did not require the presence of leg symptoms, i.e., neurogenic claudication. To identify predictors of improvement (i.e., prognostic factors) we included any study with or without a clinical intervention that included one of the outcomes we included in the current study, i.e., walking capacity and physical function and symptoms of lumbar spinal stenosis. For studies that included at least one clinical intervention and a comparison group, we also extracted information on potential treatment effect modifiers (also known as moderators), if available.

**RESULTS**

Our search strategies in Pubmed are described in **Appendix Table 1**. While piloting two initial search strategies we identified 39 and 349 potentially relevant articles for inclusion in this review. The third search strategy (lumbar stenosis AND [walking distance or Brigham or Swiss or Zurich]) yielded 697 articles through September 25, 2024. Each title/abstract was uploaded into citation management software (Zotero) and two reviewers (AF, RF) screened all articles. We identified 132 potentially relevant articles. Two authors (AR, AF, RF) reviewed the full-text version of the potentially relevant articles to identify articles that met inclusion criteria. We excluded potentially relevant articles that did not report one of our primary outcomes even if the study used other potentially relevant outcomes (e.g., Oswestry Disability Index). We excluded any cross-sectional study since these studies fail to capture improvement across two or more timepoints. The reference lists of review articles and included studies were hand searched for additional potentially relevant primary studies.

Of the 132 potentially relevant articles, we included 12 studies that reported at least one predictor or effect modifier for at least one of our primary outcomes, walking capacity and symptoms of lumbar spinal stenosis. Included studies are described in **Appendix Table 2**. We characterize the association of each predictor variable in **Appendix Table 3** including information on the direction and magnitude of association, and statistical significance. Two studies identified potential treatment effect modifiers (**Appendix Table 4)**.^1,2^

| **Appendix Table 1:** Search strategies | |
| --- | --- |
| Search | Pubmed search and results |
| Pilot search  #1 | ("Zurich Claudication Questionnaire"[All Fields] OR "Swiss Spinal Stenosis Questionnaire"[All Fields] OR ("brigham"[All Fields] AND ("spinal stenosis"[MeSH Terms] OR ("spinal"[All Fields] AND "stenosis"[All Fields]) OR "spinal stenosis"[All Fields]) AND ("questionnair"[All Fields] OR "questionnaire s"[All Fields] OR "surveys and questionnaires"[MeSH Terms] OR ("surveys"[All Fields] AND "questionnaires"[All Fields]) OR "surveys and questionnaires"[All Fields] OR "questionnaire"[All Fields] OR "questionnaires"[All Fields]))) AND ("intervention"[All Fields] OR "treatment"[All Fields] OR "conservative management"[All Fields] OR "surgery"[All Fields] OR "surgical"[All Fields] OR "non-surgical"[All Fields]) AND ("outcome"[All Fields] OR "changes"[All Fields] OR "effectiveness"[All Fields] OR "improvement"[All Fields]) AND ("predictors"[All Fields] OR "factors"[All Fields] OR "determinants"[All Fields] OR "success factors"[All Fields] OR "prognostic factors"[All Fields])  Results = 39 articles  15 selected |
| Pilot search  #2 | "lumbar spinal stenosis" AND (exercise OR "physical therapy" OR "conservative treatment" OR rehabilitation OR nonpharmacological OR non-surgical) AND (predictor OR prognosis OR "effect modifier" OR "baseline characteristics" OR demographics) AND ("walking capacity" OR pain OR "Zurich Claudication Questionnaire" OR "Spinal Stenosis Questionnaire" OR "Oswestry Disability Index" OR "Roland Morris Disability Questionnaire")  Results = 349 articles  31 new selected |
| Final  search | (lumbar stenosis) AND (walking distance or Brigham or Swiss or Zurich)  Results = 697 articles  85 new selected |
| Total | 132 unique articles (131 articles + 1 from hand search) |

| **Appendix Table 2.** Overview of studies evaluating predictors and/or effect modifiers of lumbar spinal stenosis (LSS) symptoms, function and walking capacity | | | | | | | |
| --- | --- | --- | --- | --- | --- | --- | --- |
| Author  Year  (country) | n | Mean  age | %  female | LSS diagnosis on Imaging  with/without leg symptoms | Intervention | Predictors | Relevant^a^  outcome |
| Fokter & Yerby,^2^  2006  (Slovenia) | 58 | 66 | 64 | LSS confirmed by myelography and CT/MRI AND symptoms of degenerative lumbar spinal stenosis (no inclusion criteria specific to leg symptoms) | decompressive surgery with/without fusion | Age, gender, and pre-operative symptoms and function on ZCQ | Symptom severity and physical function on ZCQ |
| Katz  et al.,^3^  1999  (USA) | 199 | 69 | 59 | LSS confirmed by CT; myelography and CT, or MRI | decompressive surgery with/without fusion | Age, comorbid health conditions, walking capacity and stenosis symptoms on Stucki questionnaire, social support, depression | Symptom severity and physical function on Stucki questionnaire |
| Ozaki  et al.,^4^  2020  (Japan) | 160 | 69 | 79 | LSS confirmed by MRI or myelography AND self-reported leg pain and neurogenic claudication | Posterior lumbar spinal surgery | Knee osteoarthritis | Symptom severity and physical function on ZCQ |
| Schneider  et al.,^5^  2016  (USA) | 59 | 62 | 33 | LSS confirmed by MRI AND self-reported leg pain for at least 6 months duration | 3 received varying dosages of distraction manipulation;  1 received placebo | Age, BMI, SSSQ total score, treadmill test, leg pain VAS, LBP VAS, duration of leg pain, ODI | Symptom severity and physical function on SSSQ (total) |
| Shi  et al.,^6^  2023  (China) | 82 | 69 | 61 | LSS confirmed by MRI; AND Self-reported leg symptoms | supervised  therapeutic exercise  3x per wk for 6 wks | Type 2 diabetes | walking capacity  on SPWT |
| Sinikallio  et al.,^7^  2007  (Finland) | 99 | 62 | 58 | LSS confirmed by myelography, CT, or MRI; AND Self-reported severe back, buttock, and/or lower extremity pain; AND surgeon’s clinical evaluation | Surgical decompression | Age, gender, marital status, comorbidity, back-related disability, back pain, previous lumbar surgery, depression, Stucki questionnaire | Symptom severity on Stucki questionnaire |
| Sinikallio  et al.,^8^  2010  (Finland) | 93 | 62 | 59 | LSS confirmed by CT, MRI, myelography; AND Self-reported severe back, buttock, and/or lower extremity pain; AND a surgeon’s clinical evaluation | Decompressive surgery | Age, gender, comorbidity, marital status, depression, pain, “misery” | Symptom severity on Stucki questionnaire |
| Sugimoto  et al.,^9^  2024  (Japan) | 209 | 76 | 47 | LSS confirmed by MRI, CT, or myelography | Lumbar decompression | Frailty | Symptom severity and physical function on ZCQ |
| Takenaka  et al.,^10^  2019  (Japan) | 78 | 70 | 44 | LSS confirmed by MRI, CT, or myelography; AND Self-reported severe leg pain and/or numbness | decompression surgery and/or fusion surgery | Age, sex, height, body weight, smoking, trunk flexor and extensor mm strength, baseline walking capacity, LBP VAS, Leg pain VAS, leg numbness VAS, area of the dural sac | walking capacity  on 6MWD |
| Thornes  et al.,^11^  2011  (Norway) | 100 | 68 | 55 | LSS confirmed by MRI; AND referred to surgical assessment | Laminectomy with or without fusion | Age, gender | Symptom severity and physical function on SSSQ |
| Turner  et al.,^1^  2015  (USA) | 400 | 68 | 55 | LSS confirmed by CT or MRI; AND Self-reported buttock/leg pain worse than back pain; AND RDQ physical disability score > 7 | Epidural injection of corticosteroid + lidocaine vs. lidocaine only | Age, gender, race, ethnicity, BMI, work status, education level, diabetes, smoking history, duration of pain, stenosis severity, treatment expectations, depressive symptoms, fear or anxiety | Symptom severity and physical function on SSSQ |
| Weiner  et al.,^12^  2021  (USA) | 193 | 66 | 3 | LSS confirmed by MRI; AND Self-reported pain, weakness, or tingling in legs with walking/ standing for at least 3 months | Decompressive laminectomy | Baseline symptoms and function on BSS, less treatment credibility and expectations, non-current smoking, no use of nonopioid pain medications | Symptom severity and physical function on BSS |
| MRI: Magnetic resonance imaging; CT: Computed Tomography; 6MWD: 6-minute walk distance; SPWT: self-paced walking test; SSSQ: Swiss spinal stenosis questionnaire; ZCQ: Zurich Claudication Questionnaire; BSS: Brigham Spinal Stenosis Questionnaire  ^a^ We only extracted data on predictors of one of our primary outcomes, walking capacity and symptoms of lumbar spinal stenosis | | | | | | | |

| **Appendix Table 3.** Predictors of lumbar spinal stenosis (LSS) symptoms, function and walking capacity | | | |
| --- | --- | --- | --- |
| Author  year | Predictor | Outcome Measure | Association  (direction, magnitude, statistical significance) |
|  | **Age** |  |  |
| Fokter & Yerby,^2^  2006 | <65, >65 years, categorical | Symptom severity and physical function  on ZCQ | Age >65 yrs was associated with less percent change in physical function score change (26.8±34.8) vs <65 yrs (43.0+35.0), although not statistically significant P =0.092  Age >65 yrs was associated with less percent change in stenosis symptom score change (26.4+36.4) vs <65 yrs (39.7±32.8), although not statistically significant P =0.168 |
| Schneider  et al.,^5^  2016 | Years, continuous | Symptom severity and physical function on SSSQ (total) | Older age versus younger was significantly associated with higher SSSQ score at completion of therapy, (adjusted)*B* = 0.26 +0.09, P= 0.026 |
| Sinikallio  et al.,^7^  2007 | Years, continuous | Symptom severity on Stucki questionnaire | Older age was associated with higher 3-month postoperative Stucki severity score (>2.4) although not statistically significant, OR 1.03, 95%CI 0.98–1.09 |
| Sinikallio  et al.,^8^  2010 | Years, continuous | Symptom severity on Stucki questionnaire | Older age was significantly associated with higher 2-year symptom severity score (> 2.4), OR 1.10, 95%CI 1.03-1.18, P< 0.01 |
| Takenaka  et al.,^10^  2019 | Years, continuous | walking capacity  on 6MWD | Older age versus younger significantly predicted less distance for postoperative 6-month walking capacity, (adjusted)B= -0.45 +0.09, P< 0.001 |
| Thornes  et al.,^11^  2011 | <65, >65 years | Symptom severity and physical function on SSSQ | Older age was associated with less physical function (P= 0.947), at each measured time point, although not statistically significant  Older age was associated with higher symptom severity (P= 0.201), at each measured time point, although not statistically significant |
| Turner  et al.,^1^  2015 | Years, continuous | Symptom severity and physical function on SSSQ | Not associated with either outcome at 3 or 6 weeks. |
| Katz  et al.,^3^  1999 | Years, continuous | Symptom severity and physical function on Stucki questionnaire | Older age was associated with less physical function at 24 months postoperative, *B=* 0.14, although not statistically significant  Older age was associated with higher symptom severity at 24 months postoperative, *B=* 0.06, although not statistically significant |
|  | **Sex/Gender** |  |  |
| Fokter & Yerby,^2^  2006 | Male, Female | Symptom severity and physical function  on ZCQ | Female gender was associated with less percent change in physical function score change (31.1±34.9) vs male gender (36.2+37.1), although not statistically significant P =0.608  Female gender was associated with less percent change in stenosis symptom score change (28.0+34.4) vs male gender (37.5±37.1), although not statistically significant P =0.332 |
| Sinikallio  et al.,^7^  2007 | Male, Female | Symptom severity on Stucki questionnaire | Male gender was predictive of higher 3-month postoperative Stucki severity (>2.4) although not statistically significant, OR 1.51 95%CI 0.53–4.30 |
| Sinikallio  et al.,^8^  2010 | Male, Female | Symptom severity on Stucki questionnaire | Male gender was predictive of higher 2-year Stucki severity (>2.4) although not statistically significant, OR 2.75, 95%CI 0.76 – 9.91 |
| Thornes  et al.,^11^  2011 | Male, Female | Symptom severity and physical function on SSSQ | Female gender was associated with less physical function (P= 0.552), at each measured time point, although not statistically significant  Female gender was associated with higher symptom severity (P= 0.605), at each measured time point, although not statistically significant |
| Turner  et al.,^1^  2015 | Male, Female | Symptom severity and physical function on SSSQ | Not associated with either outcome at 3 or 6 weeks. |
| Takenaka  et al.,^10^  2019 | Women, Men | walking capacity  on 6MWD | Sex was not associated with postoperative 6-month walking capacity, (crude) *B*=0.35 +0.11, p< 0.01 |
|  | **Race/Ethnicity** |  |  |
| Turner  et al.,^1^  2015 | Caucasian, non-Caucasian | Symptom severity and physical function on SSSQ | Non-Caucasians (vs. Caucasians) had better outcomes at 6 weeks on the physical function scale, adjusted mean difference -0.16 (-0.30, -0.01), p=0.03 |
|  | **Marital status** |  |  |
| Sinikallio  et al.,^7^  2007 | Single (no/yes) | Symptom severity on Stucki questionnaire | Marital status was predictive of higher 3-month postoperative Stucki severity (>2.4) although not statistically significant, OR 1.40, 95%CI 0.42–4.65 |
| Sinikallio  et al.,^8^  2010 | Single (yes/no) | Symptom severity on Stucki questionnaire | Marital status was significantly predictive of higher 2-year Stucki severity (>2.4), OR 6.76, 95%CI 1.46 – 31.26, P< 0.05 |
|  | **Employment/Education** |  |  |
| Turner  et al.,^1^  2015 | Employment (full-/part-time, retired not disabled, retired disabled, other), categorical | Symptom severity and physical function on SSSQ | Patients who were retired (vs. working) had significantly worse symptom severity scores at 6 weeks, adjusted mean difference 0.15 (-0.01, 0.30) and 0.28 (0.05, 0.51), p= 0.05 |
| Turner  et al.,^1^  2015 | Education level (HS or less, some college, college, prof/graduate degree), categorical | Symptom severity and physical function on SSSQ | Not associated with either outcome at 3 or 6 weeks. |
|  | **Height/Weight/BMI** |  |  |
| Takenaka  et al.,^10^  2019 | Height (cm), continuous | walking capacity  on 6MWD | Increased height was not associated with postoperative 6-month walking capacity, (crude) B=0.53 +0.10, P<0.001 |
| Takenaka  et al.,^10^  2019 | Body weight (kg), continuous | walking capacity  on 6MWD | Higher body weight predicted significantly less distance for postoperative 6MWD at 6 months, (adjusted)B= -0.20 +0.1, P< 0.05 |
| Schneider  et al.,^5^  2016 | BMI, continuous | Symptom severity and physical function on SSSQ (total) | Direction and magnitude of association not reported. No analysis data reported. |
| Turner  et al.,^1^  2015 | BMI, continuous | Symptom severity and physical function on SSSQ | Not associated with either outcome at 3 or 6 weeks |
|  | **Smoking status** |  |  |
| Weiner  et al.,^12^  2021 | Smoking status (current, prior, nonsmoker), categorical | Symptom severity and physical function on BSS | Current smoker more likely to have clinically significant improvement in physical function compared to non-smokers (OR=2.36. 95% CI: 0.95-5.83, p-value= 0.06). No association among prior smokers. No association between smoking status with clinically significant improvements in symptom severity. |
| Turner  et al.,^1^  2015 | Smoking history (never/former, current), categorical | Symptom severity and physical function on SSSQ | Not associated with either outcome at 3 or 6 weeks. |
| Takenaka  et al.,^10^  2019 | Current smoking (yes,no), categorical | Walking capacity on 6MWD | Smoking was not associated with postoperative 6-month walking capacity, (crude)B=0.23 +0.12, P= 0.04 |
|  | **Quality of life** |  |  |
| Turner  et al.,^1^  2015 | EQ-5D questionnaire, continuous  FABQ-PA questionnaire, continuous  PCS (total), continuous | Symptom severity and physical function on SSSQ | Patients with higher (better) EQ-5D index scores had significantly lower physical function scores at 6 weeks, adjusted ANCOVA coefficient -0.36 (-0.71, -0.00), p< 0.05 |
| Schneider  et al.,^5^  2016 | ODI, continuous | Symptom severity and physical function on SSSQ (total) | Not associated with outcomes. No analysis data reported. |
| Sinikallio  et al.,^7^  2007 | Baseline ODI > median (44):no/yes | Symptom severity on Stucki questionnaire | Higher disability score was predictive of higher 3-month postoperative Stucki severity (>2.4), OR 0.54, 95%CI 0.14–2.17 |
|  | **Depression** |  |  |
| Sinikallio  et al.,^7^  2007 | Baseline BDI score, continuous | Symptom severity on Stucki questionnaire | Higher baseline depression score significantly predicted higher 3-month postoperative Stucki severity (>2.4), OR 1.16, 95%CI 1.02–1.31, P< 0.05 |
| Sinikallio  et al.,^8^  2010 | 3-month BDI score continuous | Symptom severity on Stucki questionnaire | Postoperative BDI score significantly predicted higher 2-year Stucki severity (>2.4), OR 1.16, 95%CI 1.02-1.31, P< 0.05 |
| Sinikallio  et al.,^8^  2010 | 3-month Misery = VAS over median (20) and BDI >10 (no/yes), categorical | Symptom severity on Stucki questionnaire | Belonging to the postoperative misery group significantly predicted worse symptom severity (>2.4) at 2 years, OR 21.09, 95%CI 3.91 – 113.67, P< 0.001 |
| Turner  et al.,^1^  2015 | PHQ-8, continuous  GAD-7, continuous | Symptom severity and physical function on SSSQ | Patients with more severe depressive symptoms on PHQ-8 at baseline had slightly worse symptom severity scores at 6 weeks, ANCOVA coefficient 0.01 (0.00, 0.03), p= 0.04 |
| Katz  et al.,^3^  1999 | Social support scale, continuous  3-item depression scale, continuous | Symptom severity and physical function on Stucki questionnaire | Lower reported social support was associated with worse physical function (walking capacity), *B=* 0.03 and increased symptom severity, *B=* 0.02, although not statistically significant  Higher depression scale scores were significantly associated with worse physical function (walking capacity), *B=* 0.19 (P< 0.005) and increased symptom severity, *B=* 0.29 (P< 0.0005) |
|  | **Other chronic conditions** |  |  |
| Ozaki  et al.,^4^  2020 | Clinical and radiographic knee osteoarthritis, Kellgren-Lawrence grade ≥ 2 (yes, no) | Symptom severity and physical function on ZCQ | Postoperative ZCQ scores exhibited no significant difference knee OA vs control.  PF: 1.8 ± 0.6 vs 1.7 ± 0.6, P= 0.167  SS: 2.6 ± 0.9 vs 2.3 ± 0.8, P= 0.113  Pre-op vs post-op change was significant in both groups. |
| Shi  et al.,^6^  2023 | Type 2 diabetes diagnosed using American Diabetes Association criteria  (yes, no) | walking capacity  on SPWT | Patient with diabetes achieved significantly lower increase in walking capacity at 6 weeks (SPWT change 129 +251 vs 168 +150, P< 0.001) and 12 weeks (SPWT change 220 +282 vs 248 +345, P< 0.001) |
| Sugimoto  et al.,^9^  2024 | Frailty on the mFI-11 (frail(F), robust(R), pre-frail(P)) | Symptom severity and physical function on ZCQ | Post-operative ZCQ improved significantly, regardless of the degree of frailty when comparing pre-op, 6mo, and 1 yr (Kruskal–Wallis; F vs. P vs. R; SS = 0.042, 0.136, 0.059, PF = 0.004, 0.002, 0.055) |
| Katz  et al.,^3^  1999 | Cumulative Illness Rating Scale, scored 0-4 for each of 13 organ systems and overall, continuous | Symptom severity and physical function on Stucki questionnaire | Greater cardiovascular comorbidity was significantly associated with worse physical function (walking capacity), *B=* 0.30 (P< 0.0005) and increased symptom severity, *B=* 0.24 (P< 0.005)  Higher musculoskeletal comorbidity was associated with worse physical function (walking capacity), *B=* 0.17 and increased symptom severity, *B=* 0.14, although not statistically significant  Higher overall comorbidity was significantly associated with worse physical function (walking capacity), *B=* 0.33 (P< 0.0005) and increased symptom severity, *B=* 0.27 (P< 0.0005) |
| Sinikallio  et al.,^7^  2007 | Somatic comorbidity (>median (5) no/yes) | Symptom severity on Stucki questionnaire | Higher somatic comorbidity was predictive of higher 3-month postoperative Stucki severity (>2.4), although not statistically significant, OR 1.02, 95%CI 0.34–3.11 |
| Sinikallio  et al.,^8^  2010 | Somatic comorbidity (>median (5) no/yes) | Symptom severity on Stucki questionnaire | Higher somatic comorbidity was predictive of higher 2-year Stucki severity (>2.4), although not statistically significant, OR 0.69, 95%CI 0.20 – 2.40 |
|  | **Lumbar surgery** |  |  |
| Sinikallio  et al.,^7^  2007 | Previous lumbar spine surgery (no/yes), categorical | Symptom severity on Stucki questionnaire | Previous lumbar surgery predicted higher 3-month postoperative Stucki severity (>2.4), although not statistically significant, OR 6.93, 95%CI 0.63–75.96 |
|  | **Walking capacity** |  |  |
| Takenaka  et al.,^10^  2019 | Baseline 6MWD, continuous | walking capacity  on 6MWD | Lower baseline 6MWD predicted significantly less walking capacity postoperative 6MWD at 6 months, (adjusted)B= 0.31 +0.08, P< 0.001 |
| Schneider  et al.,^5^  2016 | Treadmill test  (Total minutes walked, Unilateral/bilateral leg pain, proximal/distal leg pain, qualitative description of leg symptoms) | Symptom severity and physical function on SSSQ (total) | Qualitative description as “Other” symptoms—which included any  1 of the other 6 qualitative descriptions of leg pain significantly predicted higher SSSQ at completion of care (adjusted) *B=*0.27 +2.58, P = 0.013 |
|  | **Duration of pain** |  |  |
| Turner  et al.,^1^  2015 | Duration of back or leg pain (<3 months, 3-12months, 1-5years, > 5years), categorical | Symptom severity and physical function on SSSQ | Patients with pain lasting 1 to 5 years (vs. <3months) had significantly worse symptom severity scores at 6 weeks, adjusted mean difference 0.25 (0.03, 0.47), p= 0.04. |
| Weiner  et al.,^12^  2021 | Duration of back pain, months, continuous | Symptom severity and physical function on BSS | Not associated with either outcome. |
| Schneider  et al.,^5^  2016 | Duration of leg pain (months), continuous | Symptom severity and physical function on SSSQ (total) | Not associated with outcomes. No analysis data reported |
|  | **Pain intensity** |  |  |
| Sinikallio  et al.,^8^  2010 | 3-month postoperative VAS score, continuous  3-month pain drawing markings > median(17) (no/yes), categorical | Symptom severity on Stucki questionnaire | Postoperative VAS score significantly predicted higher 2-year Stucki severity (>2.4), OR 1.06, 95%CI 1.02-1.10, P< 0.01  Postoperative pain drawing was predictive of higher 2-year Stucki severity (>2.4), although not statistically significant, OR 1.21, 95%CI 0.38 – 3.81 |
| Takenaka  et al.,^10^  2019 | LBP VAS, leg pain VAS, leg numbness VAS, continuous (each) | Walking capacity on 6MWD | Back pain VAS was not associated with postoperative 6-month walking capacity, (crude)B= 0.10 +0.12, P= 0.42  Leg pain VAS was not associated with postoperative 6-month walking capacity, (crude)B= 0.27 +0.11, P< 0.05  Leg numbness VAS was not associated with postoperative 6-month walking capacity, (crude)B= 0.21 +0.11, P= 0.08 |
| Sinikallio  et al.,^7^  2007 | Baseline VAS score > median (61), no/yes, categorical | Symptom severity on Stucki questionnaire | Higher VAS score was predictive of higher 3-month postoperative Stucki severity (>2.4), although not statistically significant, OR 2.53, 95%CI 0.70–9.17 |
| Schneider  et al.,^5^  2016 | Baseline severity, leg pain and back pain, VAS scale, continuous | Symptom severity and physical function on SSSQ (total) | Not associated with outcomes. No analysis data reported |
|  | **Stenosis symptoms** |  |  |
| Fokter & Yerby,^2^  2006 | Pre-Op SS score (<4.0, >4.0), categorical | Symptom severity and physical function  on ZCQ | Less severe pre-operative symptoms was associated with lower percent change (28.3±37.1) PF score versus more severe pre-operative symptoms (37.7±33.8), P= 0.319  Less severe pre-operative symptoms was significantly associated with lower percent change (19.5±35.4) SS score versus more severe pre-operative symptoms (43.4±31.6), P= 0.009 |
| Fokter & Yerby,^2^  2006 | Pre-Op PF score (<3.4, >3.4), categorical | Symptom severity and physical function  on ZCQ | Better baseline physical function was associated with lower percent change (26.4±38.1) PF score versus worse baseline physical function (37.3+33.5), P= 0.257  Better baseline physical function was significantly associated with lower percent change (20.6±40.1) PF score versus worse baseline physical function (38.6+30.4), P= 0.057 |
| Schneider  et al.,^5^  2016 | Baseline SSSQ  (total score, neuro-ischemic subscore, symptom severity subscore), continuous | Symptom severity and physical function on SSSQ (total) | Higher baseline SSSQ score was significantly associated with a higher SSSQ score at completion of care, (adjusted) *B=*0.50 +4.54, p< 0.001 |
| Weiner  et al.,^12^  2021 | Baseline BSS physical function, continuous | Symptom severity and physical function on BSS | Higher physical function baseline scores (per 0.2 point) associated with an increased odds of clinically significant physical function change (OR=1.24, 95%CI= 1.11-1.38, p<0.001) and symptom severity change (OR=1.14, 95%CI= 1.02-1.26, p=0.016). |
| Weiner  et al.,^12^  2021 | Baseline BSS symptom severity, continuous | Symptom severity and physical function on BSS | Higher severity scores associated with clinically significant improvement in symptom severity (OR=1.18, 95%CI= 1.07-1.30, p <0.001) but no physical function |
| Sinikallio  et al.,^7^  2007 | Baseline Stucki severity >median (3.3), no/yes, categorical | Symptom severity on Stucki questionnaire | Higher Stucki severity at baseline was predictive of higher 3-month postoperative Stucki severity (>2.4), although not statistically significant, OR 2.93, 95%CI 0.87–9.86 |
| Turner  et al.,^1^  2015 | Categorical (mild, moderate, severe) | Symptom severity and physical function on SSSQ | Not associated with either outcome at 3 or 6 weeks. |
| Katz  et al.,^3^  1999 | Baseline physical function (walking capacity) on Stucki questionnaire, continuous | Symptom severity and physical function on Stucki questionnaire | Decreased physical function(walking capacity) at baseline was significantly associated with decreased physical function at 24 months postoperative, *B=* 0.30 (P< 0.0005)  Decreased physical function(walking capacity) at baseline was significantly associated with increased symptom severity at 24 months postoperative, *B=* 0.20 (P< 0.005) |
| Katz  et al.,^3^  1999 | Baseline symptom severity on Stucki questionnaire, continuous | Symptom severity and physical function on Stucki questionnaire | Increased symptom severity at baseline was significantly associated with decreased physical function at 24 months postoperative, *B=* 0.24 (P< 0.0005)  Increased symptom severity at baseline was significantly associated with increased symptom severity at 24 months postoperative, *B=* 0.25 (P< 0.005) |
|  | **Other variables** |  |  |
| Takenaka  et al.,^10^  2019 | Area of dural sac (mm^2^), continuous | Walking capacity on 6MWD | Dural sac measurement was not associated with postoperative 6-month walking capacity, (crude)B= 0.43 +0.11, P< 0.001 |
| Takenaka  et al.,^10^  2019 | Trunk flexor mm strength, extensor mm strength | Walking capacity on 6MWD | Flexor muscle strength was not associated with postoperative 6-month walking capacity, (crude)B= 0.44 + 0.10, P< 0.001  Extensor muscle strength significantly predicted postoperative 6-month walking capacity, (adjusted)B= 0.26 +0.11, P< 0.05 |
| 6MWD: 6-minute walk distance; SPWT: self-paced walking test; SSSQ: Swiss spinal stenosis questionnaire; ZCQ: Zurich Claudication Questionnaire; BSS: Brigham Spinal Stenosis Questionnaire | | | |

| **Appendix Table 4.** Potential treatment effect modifiers of of lumbar spinal stenosis (LSS) symptoms, function and walking capacity | | |
| --- | --- | --- |
| Author  year | Outcome Measure | Key findings |
|  |  |  |
| Turner  et al.,^1^  2015 | Symptom severity and physical function on SSSQ | Patients in the ‘‘other’’ employment category (i.e., not working or retired) had larger treatment effects on SSSQ Function scores at 6 weeks if assigned to lidocaine only versus corticosteroid + lidocaine (p-for-interaction=0.02)  No baseline variable modified the effect of treatment on the SSSQ Symptom Severity score  (See above in Appendix Table 2 for list of variables that were evaluated as potential effect modifiers) |
| Fokter & Yerby,^2^  2006 | Symptom severity and physical function  on ZCQ | The mean SS domain score change and the percent change, were significantly greater for the fusion group.  The operative group with three/four levels had a significantly lower percent change in the PF domain score (0.62±0.85) versus one/two levels (1.02±0.73) |

Reference List

1. Turner JA, Comstock BA, Standaert CJ, et al. Can patient characteristics predict benefit from epidural corticosteroid injections for lumbar spinal stenosis symptoms? *Spine J*. Nov 1 2015;15(11):2319-31. doi:10.1016/j.spinee.2015.06.050

2. Fokter SK, Yerby SA. Patient-based outcomes for the operative treatment of degenerative lumbar spinal stenosis. *Eur Spine J*. Nov 2006;15(11):1661-9. doi:10.1007/s00586-005-0033-4

3. Katz JN, Stucki G, Lipson SJ, Fossel AH, Grobler LJ, Weinstein JN. Predictors of surgical outcome in degenerative lumbar spinal stenosis. *Spine (Phila Pa 1976)*. Nov 1 1999;24(21):2229-33. doi:10.1097/00007632-199911010-00010

4. Ozaki M, Fujita N, Miyamoto A, et al. Impact of knee osteoarthritis on surgical outcomes of lumbar spinal canal stenosis. *J Neurosurg Spine*. May 1 2020;32(5):710-715. doi:10.3171/2019.10.SPINE19886

5. Schneider MJ, Terhorst L, Murphy D, Stevans JM, Hoffman R, Cambron JA. Exploratory Analysis of Clinical Predictors of Outcomes of Nonsurgical Treatment in Patients With Lumbar Spinal Stenosis. *J Manipulative Physiol Ther*. Feb 2016;39(2):88-94. doi:10.1016/j.jmpt.2016.01.001

6. Shi T, Chen Z, Hu D, Li W, Wang Z, Liu W. Does type 2 diabetes affect the efficacy of therapeutic exercises for degenerative lumbar spinal stenosis? *BMC Musculoskelet Disord*. Mar 16 2023;24(1):198. doi:10.1186/s12891-023-06305-0

7. Sinikallio S, Aalto T, Airaksinen O, et al. Depression is associated with poorer outcome of lumbar spinal stenosis surgery. *Eur Spine J*. Jul 2007;16(7):905-12. doi:10.1007/s00586-007-0349-3

8. Sinikallio S, Aalto T, Lehto SM, et al. Depressive symptoms predict postoperative disability among patients with lumbar spinal stenosis: a two-year prospective study comparing two age groups. *Disabil Rehabil*. 2010;32(6):462-8. doi:10.3109/09638280903171477

9. Sugimoto S, Nagai S, Ito K, et al. The Impact of Frailty on Surgical Outcome of Patients with Lumbar Spinal Canal Stenosis. *Spine Surg Relat Res*. Mar 27 2024;8(2):188-194. doi:10.22603/ssrr.2023-0171

10. Takenaka H, Sugiura H, Kamiya M, et al. Predictors of walking ability after surgery for lumbar spinal canal stenosis: a prospective study. *Spine J*. Nov 2019;19(11):1824-1831. doi:10.1016/j.spinee.2019.07.002

11. Thornes E, Ikonomou N, Grotle M. Prognosis of surgical treatment for degenerative lumbar spinal stenosis: a prospective cohort study of clinical outcomes and health-related quality of life across gender and age groups. *Open Orthop J*. 2011;5:372-8. doi:10.2174/1874325001105010372

12. Weiner DK, Holloway K, Levin E, et al. Identifying biopsychosocial factors that impact decompressive laminectomy outcomes in veterans with lumbar spinal stenosis: a prospective cohort study. *Pain*. Mar 1 2021;162(3):835-845. doi:10.1097/j.pain.0000000000002072

**Additional tables**

| **Appendix Table 5.** Predictors of 2-month change in lumbar spinal stenosis symptom severity and function on the total score of the Swiss Spinal Stenosis Questionnaire (SSSQ) | | | | |
| --- | --- | --- | --- | --- |
|  |  | SSSQ total score | | |
| Baseline characteristic | n | Baseline  mean ±Std | 2-month change  mean ±Std | 2-month unadjusted  mean difference (95%CI) |
| Total sample | 216 | 31.3 ±5.9 | -2.5 ±5.6 | n/a |
| Age |  |  |  |  |
| <70 | 100 | 32.1 ±5.5 | -3.2 ±5.7 | Ref |
| >70 | 116 | 30.7 ±6.2 | -2.0 ±5.6 | 1.10 (-0.37 to 2.70) |
| Sex |  |  |  |  |
| Male | 100 | 31.4 ±6.1 | -2.4 ±5.4 | 0.30 (-1.24 to 1.79) |
| Female | 116 | 31.3 ±5.7 | -2.7 ±5.8 | Ref |
| Race |  |  |  |  |
| White | 165 | 30.9 ±6.1 | -2.1 ±5.7 | Ref |
| Non-White | 50 | 32.8 ±4.9 | -3.8 ±5.1 | -1.73 (-3.51 to 0.05) |
| Household income |  |  |  |  |
| ≥$40,000/y | 102 | 30.7 ±5.9 | -2.0 ±5.6 | Ref |
| <40,000/y | 106 | 32.1 ±5.6 | -3.2 ±5.6 | -1.22 (-2.76 to 0.32) |
| Relationship status |  |  |  |  |
| Married | 112 | 30.7 ±6.3 | -2.7 ±5.7 | Ref |
| Not married | 104 | 32.0 ±5.4 | -2.4 ±5.6 | 0.22 (-1.29 to 1.73) |
| Body mass index |  |  |  |  |
| ≤30 | 115 | 30.9 ±6.2 | -2.5 ±5.1 | Ref |
| >30 | 101 | 31.8 ±5.5 | -2.7 ±6.2 | -0.20 (-1.72 to 1.31) |
| Smoking status |  |  |  |  |
| Never smoker | 92 | 31.1 ±5.3 | -3.5 ±5.3 | Ref |
| Former or current smoker | 120 | 31.6 ±6.3 | -1.9 ±5.8 | 1.60 (0.07 to 3.14) |
| Duration of back symptoms |  |  |  |  |
| ≤6 months | 22 | 28.5 ±5.3 | -0.6 ±6.5 | 2.13 (-0.36 to 4.61) |
| >6 months | 194 | 31.7 ±5.9 | -2.8 ±5.5 | Ref |
| Duration of leg symptoms |  |  |  |  |
| ≤6 months | 58 | 29.7 ±5.8 | -2.3 ±6.4 | 0.32 (-1.38 to 2.03) |
| >6 months | 158 | 31.9 ±5.8 | -2.6 ±5.4 | Ref |
| Diagnostic imaging results |  |  |  |  |
| Central canal stenosis only | 17 | 31.4 ±5.1 | -2.3 ±6.0 | 0.27 (-2.66 to 3.20) |
| Lateral canal stenosis only | 88 | 31.4 ±6.3 | -2.4 ±5.4 | 0.20 (-1.42 to 1.82) |
| Central and lateral stenosis | 103 | 31.3 ±5.9 | -2.6 ±5.8 | Ref |
| Hip osteoarthritis |  |  |  |  |
| Yes | 35 | 32.7 ±5.6 | -2.4 ±6.0 | Ref |
| No | 181 | 31.1 ±5.9 | -2.6 ±5.6 | -0.21 (-2.26 to 1.84) |
| Knee osteoarthritis |  |  |  |  |
| Yes | 148 | 30.7 ±5.5 | -2.3 ±5.7 | 0.66 (-0.96 to 2.29) |
| No | 68 | 32.8 ±6.5 | -3.0 ±5.5 | Ref |
| No. of comorbidities |  |  |  |  |
| ≤4 | 117 | 30.5 ±5.9 | -2.1 ±5.2 | Ref |
| >4 | 99 | 32.3 ±5.8 | -3.1 ±6.0 | -1.04 (-2.55 to 0.47) |
| Ankle-brachial index |  |  |  |  |
| <1 | 77 | 31.3 ±6.2 | -2.4 ±5.4 | 0.20 (-1.38 to 1.78) |
| ≥1 | 139 | 31.4 ±5.7 | -2.6 ±5.8 | Ref |
| SSSQ total score |  |  |  |  |
| < 31 | 109 | 26.8 ±3.6 | -0.9 ±5.3 | Ref |
| > 31 | 107 | 36.0 ±3.8 | -4.2 ±5.4 | -3.27 (-4.72 to -1.82) |
| Walking distance on SPWT |  |  |  |  |
| < 280 | 111 | 32.8 ±5.9 | -2.9 ±5.6 | -0.80 (-2.31 to 0.71) |
| > 280 | 105 | 29.8 ±5.5 | -2.1 ±5.6 | Ref |
| Oswestry Disability Index |  |  |  |  |
| ≤40 | 128 | 29.1 ±5.3 | -1.9 ±5.5 | Ref |
| >40 | 88 | 34.7 ±5.1 | -3.4 ±5.7 | -1.48 (-3.00 to 0.05) |
| Leg pain intensity |  |  |  |  |
| ≤6 | 131 | 29.6 ±5.2 | -2.2 ±4.9 | Ref |
| >6 | 85 | 34.0 ±5.9 | -3.1 ±6.6 | -0.98 (-2.62 to 0.66) |
| Back pain intensity |  |  |  |  |
| ≤6 | 94 | 29.4 ±5.2 | -2.1 ±5.2 | Ref |
| >6 | 122 | 32.8 ±6.0 | -2.9 ±5.9 | -0.85 (-2.38 to 0.67) |
| Gait speed |  |  |  |  |
| <1 m/s | 135 | 32.0 ±5.8 | -2.9 ±5.5 | -0.81 (-2.37 to 0.74) |
| ≥1 m/s | 81 | 30.3 ±5.9 | -2.0 ±5.8 | Ref |
| Physical activity |  |  |  |  |
| <165 min/d | 121 | 31.7 ±5.7 | -2.4 ±5.9 | 0.32 (-1.22 to 1.86) |
| ≥165 min/d | 89 | 30.7 ±6.1 | -2.7 ±5.2 | Ref |
| PROMIS Depression |  |  |  |  |
| <55 | 172 | 30.5 ±5.6 | -2.3 ±5.4 | 1.47 (-0.42 to 3.37) |
| ≥55 | 42 | 34.9 ±6.1 | -3.8 ±6.1 | Ref |
| Tampa Scale of Kinesiophobia |  |  |  |  |
| <26 | 103 | 30.6 ±5.1 | -2.8 ±5.3 | -0.46 (-1.97 to 1.05) |
| ≥26 | 113 | 32.0 ±6.5 | -2.3 ±5.9 | Ref |
| Treatment expectancy of MTE |  |  |  |  |
| ≤42 | 86 | 31.1 ±6.2 | -1.7 ±5.5 | Ref |
| >42 | 130 | 31.5 ±5.7 | -3.1 ±5.7 | -1.49 (-3.02 to 0.05) |

| **Appendix Table 6.** Predictors of 2-month change in walking distance on the self-paced walking test* | | | | |
| --- | --- | --- | --- | --- |
|  |  | Walking distance, meters | | |
| Baseline characteristic | N | Baseline,  mean ±Std | 2-month change  mean ±Std | 2-month unadjusted  mean difference (95%CI) |
| Total sample | 216 | 451 ±465 | 205 ±472 | n/a |
| Age |  |  |  |  |
| <70 | 100 | 501 *±*508 | 283 ±511 | 146 (20 to 271) |
| >70 | 116 | 408 ±422 | 138 ±427 | Reference |
| Sex |  |  |  |  |
| Male | 100 | 493 ±479 | 252 ±485 | 88 (-38 to 215) |
| Female | 116 | 414 ±452 | 164 ±459 | Reference |
| Race |  |  |  |  |
| White | 165 | 477 ±492 | 197 ±501 | Reference |
| Non-White | 50 | 366 ±359 | 225 ±365 | 29 (-100 to 157) |
| Household income |  |  |  |  |
| ≥$40,000/y | 102 | 482 ±490 | 193 ±490 | Reference |
| <40,000/y | 106 | 396 ±375 | 210 ±458 | 17 (-112 to 147) |
| Relationship status |  |  |  |  |
| Married | 112 | 488 ±496 | 243 ±512 | Reference |
| Not married | 104 | 411 ±428 | 164 ±424 | -79 (-206 to 47) |
| Body mass index |  |  |  |  |
| ≤30 | 115 | 526 ±507 | 249 ±515 | 94 (-31 to -219) |
| >30 | 101 | 365 ±398 | 155 ±415 | Reference |
| Smoking status |  |  |  |  |
| Never smoker | 92 | 498 ±552 | 191 ±451 | Reference |
| Former or current smoker | 120 | 417 ±392 | 208 ±482 | 17 (-111 to 145) |
| Duration of back symptoms |  |  |  |  |
| ≤6 months | 22 | 318 ±254 | 286 ±398 | 90 (-119 to 300) |
| >6 months | 194 | 466 ±481 | 196 ±480 | Reference |
| Duration of leg symptoms |  |  |  |  |
| ≤6 months | 58 | 320 ±284 | 271 ±476 | 90 (-52 to 233) |
| >6 months | 181 | 499 ±508 | 181 ±470 | Reference |
| Diagnostic imaging results |  |  |  |  |
| Central canal stenosis only | 17 | 351 ±320 | 190 ±328 | -8 (-251 to 236) |
| Lateral canal stenosis only | 88 | 472 ±528 | 199 ±470 | 1 (-134 to 136) |
| Central and lateral stenosis | 103 | 448 ±442 | 198 ±492 | Reference |
| Hip osteoarthritis |  |  |  |  |
| Yes | 35 | 408 ±363 | 294 ±642 | 106 (-123 to 335) |
| No | 181 | 459 ±483 | 188 ±432 | Reference |
| Knee osteoarthritis |  |  |  |  |
| Yes | 148 | 398 ±478 | 114 ±371 | Reference |
| No | 68 | 475 ±459 | 247 ±508 | 133 (12 to 254) |
| No. of comorbidities |  |  |  |  |
| ≤4 | 117 | 462 ±453 | 180 ±460 | Reference |
| >4 | 99 | 438 ±481 | 234 ±486 | 52 (-74 to 181) |
| Ankle-brachial index |  |  |  |  |
| <1 | 77 | 384 ±397 | 244 ±446 | 61 (-72 to 193) |
| ≥1 | 139 | 488 ±497 | 183 ±486 | Reference |
| SSSQ total score |  |  |  |  |
| < 31 | 109 | 516 ±489 | 243 ±562 | 76 (-50 to 203) |
| > 31 | 107 | 385 ±432 | 166 ±357 | Reference |
| Walking distance on SPWT |  |  |  |  |
| < 280 | 135 | 153 ±74 | 169 ±311 | Reference |
| > 280 | 81 | 765 ±497 | 243 ±596 | 73 (-56 to 202) |
| Oswestry Disability Index |  |  |  |  |
| ≤40 | 128 | 506 ±473 | 258 ±521 | Reference |
| >40 | 88 | 370 ± 444 | 127 ±381 | -131 (-252 to -11) |
| Leg pain intensity |  |  |  |  |
| ≤6 | 131 | 481 ±467 | 209 ±502 | Reference |
| >6 | 85 | 404 ±461 | 199 ±426 | -10 (-140 to 119) |
| Back pain intensity |  |  |  |  |
| ≤6 | 94 | 520 ±496 | 230 ±549 | 44 (-89 to 177) |
| >6 | 122 | 398 ±435 | 186 ±405 | Reference |
| Gait speed |  |  |  |  |
| <1 m/s | 135 | 320 ±336 | 174 ±426 | Reference |
| ≥1 m/s | 81 | 668 ±562 | 256 ±539 | 82 (-57 to 221) |
| Physical activity |  |  |  |  |
| <165 min/d | 121 | 363 ±369 | 146 ±431 | Reference |
| ≥165 min/d | 89 | 559 ±525 | 295 ±512 | 148 (20 to 277) |
| PROMIS Depression |  |  |  |  |
| <55 | 172 | 483 ±491 | 211 ±499 | 15 (-115 to 144) |
| ≥55 | 42 | 324 ±325 | 197 ±342 | Ref |
| Tampa Scale of Kinesiophobia |  |  |  |  |
| <26 | 103 | 484 ±478 | 230 ±501 | 47 (-80 to 174) |
| ≥26 | 113 | 420 ±454 | 182 ±445 | Ref |
| Treatment expectations of MTE |  |  |  |  |
| Lower expectations, ≤42 | 86 | 456 ±484 | 193 ±417 | Ref |
| Higher expectations, >42 | 130 | 448 ±454 | 213 ±507 | 20 (-110 to 150) |
| *Self-paced walking test is the distance walked before stopping due to symptoms with a maximum duration of 30 minutes. | | | | |

| **Appendix Table 7.** Treatment effect modification of Manual Therapy and Exercise (MTE) compared to Group Exercise (GE) and Medical Care (MC) for change in symptoms and function on the total score of the Swiss Spinal Stenosis Questionnaire (SSSQ) at 2 months. | | | | | | | | | | |
| --- | --- | --- | --- | --- | --- | --- | --- | --- | --- | --- |
|  |  | 2-month change of SSSQ total score, mean ± SD | | | | |  |  | 2-month unadjusted  mean difference (95%CI) | |
| Baseline characteristic | n | MTE | n | GE | n | MC | p-value† |  | MTE vs. GE/MC | p-value |
| Total sample | 75 | -3.9 ±5.8 | 65 | -1.8 ±5.2 | 76 | -1.8 ±5.6 | - |  | -2.15 (-3.71 to -0.58) | - |
| Age |  |  |  |  |  |  | 0.05 |  |  | 0.02 |
| <70 | 35 | -5.8 ±5.7 | 26 | -2.3 ±4.0 | 39 | -1.4 ±5.9 |  |  | -4.06 (-6.29 to -1.83) |  |
| >70 | 40 | -2.3 ±5.5 | 39 | -1.4 ±5.9 | 37 | -2.3 ±5.3 |  |  | -0.47 (-2.63 to 1.69) |  |
| Sex |  |  |  |  |  |  | 0.57 |  |  | 0.35 |
| Male | 30 | -3.3 ±6.0 | 35 | -2.2 ±5.6 | 35 | -1.8 ±4.7 |  |  | -1.29 (-3.63 to 1.06) |  |
| Female | 45 | -4.4 ±5.7 | 30 | -1.2 ±4.8 | 41 | -1.9 ±6.3 |  |  | -2.79 (-4.93 to 0.64) |  |
| Race |  |  |  |  |  |  | 0.80 |  |  | 0.50 |
| White | 58 | -3.4 ±6.0 | 50 | -1.5 ±5.5 | 57 | -1.4 ±5.5 |  |  | -1.93 (-3.76 to 0.11) |  |
| Non-white | 17 | -5.9 ±4.9 | 15 | -2.7 ±4.1 | 18 | -2.8 ±5.6 |  |  | -3.18 (-6.13 to 0.24) |  |
| Relationship status |  |  |  |  |  |  | 0.47 |  |  | 0.23 |
| Married | 41 | -3.4 ±6.4 | 34 | -2.3 ±5.5 | 37 | -2.1 ±5.2 |  |  | -1.24 (-3.45 to 0.97) |  |
| Not married | 34 | -4.6 ±5.2 | 31 | -1.2 ±4.9 | 39 | -1.6 ±6.0 |  |  | -3.16 (-5.40 to 0.92) |  |
| Household income |  |  |  |  |  |  | 0.29 |  |  | 0.25 |
| ≥$40,000/y | 35 | -2.8 ±6.1 | 31 | -2.0 ±4.7 | 36 | -1.2 ±5.9 |  |  | -1.23 (-3.56 to 1.10) |  |
| <40,000/y | 38 | -5.2 ±5.5 | 31 | -1.4 ±5.6 | 37 | -2.7 ±5.3 |  |  | -3.08 (-5.27 to 0.89) |  |
| Body mass index |  |  |  |  |  |  | 0.68 |  |  | 0.45 |
| ≤30 | 42 | -3.5 ±5.2 | 39 | -1.6 ±5.1 | 34 | -2.1 ±5.0 |  |  | -1.61 (-3.57 to 0.35) |  |
| >30 | 33 | -4.5 ±6.6 | 26 | -2.0 ±5.4 | 42 | -1.6 ±6.1 |  |  | -2.81 (-5.36 to 0.26) |  |
| Smoking status |  |  |  |  |  |  | 0.78 |  |  | 0.86 |
| Never smoker | 35 | -4.7 ±5.4 | 27 | -2.2 ±4.6 | 30 | -3.3 ±5.7 |  |  | -1.90 (-4.15 to 0.35) |  |
| Former or current smoker | 40 | -3.3 ±6.2 | 37 | -1.3 ±5.6 | 43 | -1.0 ±5.5 |  |  | -2.18 (-4.38 to 0.03) |  |
| Duration of back symptoms |  |  |  |  |  |  | 0.12 |  |  | 0.09 |
| ≤6 months | 6 | 1.0 ±1.7 | 10 | -0.1 ±8.3 | 6 | -3.2 ±6.0 |  |  | 2.25 (-1.94 to 6.44) |  |
| >6 months | 69 | -4.4 ±5.9 | 55 | -2.1 ±4.5 | 70 | -1.7 ±5.6 |  |  | -2.50 (-4.10 to 0.91) |  |
| Duration of leg symptoms |  |  |  |  |  |  | 0.68 |  |  | 0.90 |
| ≤6 months | 18 | -3.7 ±5.6 | 21 | -1.0 ±6.5 | 19 | -2.4 ±7.0 |  |  | -1.97 (-5.58 to 1.65) |  |
| >6 months | 57 | -4.0 ±5.9 | 44 | -2.1 ±4.5 | 57 | -1.6 ±5.1 |  |  | -2.19 (-3.92 to 0.47) |  |
| Diagnostic imaging results |  |  |  |  |  |  | 0.87 |  |  | 0.81 |
| Central canal stenosis only | 6 | -3.0 ±6.8 | 5 | -1.6 ±4.7 | 6 | -2.2 ±7.0 |  |  | -1.09 (-7.73 to 5.55) |  |
| Lateral canal stenosis only | 30 | -4.2 ±5.9 | 20 | -1.9 ±4.3 | 38 | -1.2 ±5.4 |  |  | -2.74 (-5.11 to 0.36) |  |
| Central and lateral stenosis | 36 | -3.8 ±6.0 | 36 | -1.4 ±5.7 | 31 | -2.5 ±5.7 |  |  | -1.82 (-4.20 to 0.55 |  |
| Hip osteoarthritis |  |  |  |  |  |  | 0.78 |  |  | 0.60 |
| Yes | 13 | -4.3 ±6.4 | 10 | -0.6 ±5.5 | 12 | -1.8 ±5.8 |  |  | -3.08 (-7.26 to 1.10) |  |
| No | 62 | -3.9 ±5.8 | 55 | -2.0 ±5.2 | 64 | -1.9 ±5.6 |  |  | -1.96 (-3.67 to 0.26) |  |
| Knee osteoarthritis |  |  |  |  |  |  | 0.68 |  |  | 0.40 |
| Yes | 24 | -3.8 ±5.1 | 16 | -2.9 ±4.7 | 28 | -2.4 ±6.3 |  |  | -1.16 (-3.97 to 1.65) |  |
| No | 51 | -4.0 ±6.2 | 49 | -1.4 ±5.3 | 48 | -1.5 ±5.1 |  |  | -2.60 (-4.50 to 0.69) |  |
| No. of comorbidities |  |  |  |  |  |  | 0.89 |  |  | 0.69 |
| ≤4 | 38 | -3.7 ±5.7 | 43 | -1.3 ±4.4 | 36 | -1.3 ±5.3 |  |  | -2.39 (-4.40 to 0.39) |  |
| >4 | 37 | -4.2 ±6.0 | 22 | -2.7 ±6.5 | 40 | -2.3 ±5.8 |  |  | -1.76 (-4.24 to 0.72) |  |
| Ankle-brachial index |  |  |  |  |  |  | 0.34 |  |  | 0.64 |
| <1 | 29 | -4.1 ±5.0 | 25 | -0.5 ±4.5 | 23 | -2.4 ±6.2 |  |  | -2.65 (-5.12 to 0.19) |  |
| ≥1 | 46 | -3.9 ±6.3 | 40 | -2.5 ±5.5 | 53 | -1.6 ±5.3 |  |  | -1.87 (-3.91 to 0.17) |  |
| SSSQ total score |  |  |  |  |  |  | 0.47 |  |  | 0.79 |
| < 31 | 40 | -2.5 ±5.3 | 30 | 0.8 ±4.8 | 39 | -0.6 ±5.5 |  |  | -2.49 (-4.55 to 0.42) |  |
| > 31 | 35 | -5.6 ±6.1 | 35 | -3.9 ±4.6 | 37 | -3.1 ±5.4 |  |  | -2.09 (-4.29 to 0.11) |  |
| Walking distance on SPWT |  |  |  |  |  |  | 0.86 |  |  | 0.76 |
| < 280 | 35 | -4.3 ±5.8 | 34 | -2.1 ±5.7 | 42 | -2.5 ±5.3 |  |  | -1.97 (-4.22 to 0.28) |  |
| > 280 | 40 | -3.7 ±5.9 | 31 | -1.4 ±4.7 | 34 | -1.0 ±5.9 |  |  | -2.45 (-4.66 to 0.24) |  |
| Oswestry Disability Index |  |  |  |  |  |  | 0.41 |  |  | 0.27 |
| ≤40 | 47 | -2.9 ±5.4 | 37 | -1.0 ±5.2 | 44 | -1.7 ±5.8 |  |  | -1.53 (-3.52 to 0.45) |  |
| >40 | 28 | -5.7 ±6.3 | 28 | -2.8 ±5.1 | 32 | -2.0 ±5.3 |  |  | -3.31 (-5.84 to 0.79) |  |
| Leg pain intensity |  |  |  |  |  |  | 0.29 |  |  | 0.20 |
| ≤6 | 48 | -3.1 ±4.8 | 39 | -1.2 ±5.0 | 44 | -2.0 ±4.9 |  |  | -1.42 (-3.18 to 0.33) |  |
| >6 | 27 | -5.5 ±7.1 | 26 | -2.6 ±5.5 | 32 | -1.6 ±6.5 |  |  | -3.48 (-6.45 to 0.52) |  |
| Back pain intensity |  |  |  |  |  |  | 0.04 |  |  | 0.50 |
| ≤6 | 31 | -2.3 ±5.6 | 35 | -1.1 ±4.8 | 28 | -3.0 ±5.2 |  |  | -0.34 (-2.60 to 1.93) |  |
| >6 | 44 | -5.1 ±5.8 | 30 | -2.5 ±5.7 | 48 | -1.2 ±5.7 |  |  | -3.43 (-5.58 to 1.29) |  |
| Gait speed |  |  |  |  |  |  | 0.91 |  |  | 0.80 |
| <1 m/s | 47 | -4.1 ±5.8 | 38 | -2.0 ±5.3 | 50 | -2.3 ±5.3 |  |  | -1.99 (-3.94 to 0.04) |  |
| ≥1 m/s | 28 | -3.6 ±6.0 | 27 | -1.4 ±5.2 | 26 | -1.0 ±6.1 |  |  | -2.40 (-5.07 to 0.27) |  |
| Physical activity |  |  |  |  |  |  | 0.11 |  |  | 0.12 |
| <165 min/d | 40 | -4.6 ±6.0 | 38 | -0.5 ±5.1 | 43 | -2.0 ±5.9 |  |  | -3.32 (-5.49 to 1.14) |  |
| ≥165 min/d | 34 | -3.2 ±5.7 | 24 | -3.1 ±4.1 | 31 | -1.8 ±5.4 |  |  | -0.82 (-3.08 to 1.43) |  |
| PROMIS Depression |  |  |  |  |  |  | 0.07 |  |  | 0.10 |
| <55 | 61 | -3.5 ±5.4 | 50 | -1.2 ±4.7 | 61 | -2.1 ±5.9 |  |  | -1.83 (-3.52 to -0.13) |  |
| >55 | 12 | -7.5 ±6.1 | 15 | -3.7 ±6.4 | 15 | -0.9 ±4.4 |  |  | -5.20 (-9.16 to -1.24) |  |
| Tampa Scale of Kinesiophobia |  |  |  |  |  |  | 0.63 |  |  | 0.92 |
| <26 | 39 | -4.2 ±5.1 | 32 | -1.4 ±5.0 | 32 | -2.5 ±5.6 |  |  | -2.20 (-4.31 to -0.09) |  |
| >26 | 36 | -3.7 ±6.6 | 33 | -2.1 ±5.5 | 44 | -1.4 ±5.6 |  |  | -2.05 (-4.39 to 0.30) |  |
| Treatment expectancy of MTE |  |  |  |  |  |  | 0.40 |  |  | 0.17 |
| ≤42 | 29 | -2.2 ±6.3 | 28 | -1.4 ±5.0 | 29 | -1.3 ±5.3 |  |  | -0.79 (-3.28 to 1.71) |  |
| >42 | 46 | -5.1 ±5.3 | 37 | -2.0 ±5.4 | 47 | -2.1 ±5.8 |  |  | -2.98 (-4.98 to -0.99) |  |
| * Spinal stenosis symptoms and function scores range from 12 to 52, with higher scores indicating worse symptoms and function.  † Test for interaction (i.e. statistical effect modification) between baseline characteristic and the three treatment groups | | | | | | | | | | |

| **Appendix Table 8.** Treatment effect modification of Manual Therapy and Exercise (MTE) compared to Group Exercise (GE) and Medical Care (MC) for change in walking distance on the self-paced walking* test at 2 months. | | | | | | | | | | |
| --- | --- | --- | --- | --- | --- | --- | --- | --- | --- | --- |
|  |  | 2-month change in walking distance meters, mean ± SD | | | | |  |  | 2-month unadjusted  mean difference (95%CI) | |
| Baseline characteristic |  | MTE |  | GE |  | MC | p-value† |  | MTE vs. GE/MC | p-value |
| Total sample | 75 | 268 ±508 | 65 | 219 ±413 | 76 | 131 ± 479 | - |  | 96 (-36 to 229) | - |
| Age |  |  |  |  |  |  | 0.14 |  |  | 0.07 |
| <70 | 35 | 429 ±616 | 26 | 211 ±326 | 39 | 200 ± 490 |  |  | 224 (-10 to 459) |  |
| >70 | 40 | 127 ±339 | 39 | 225 ±466 | 37 | 57 ± 462 |  |  | -16 (-167 to 134) |  |
| Sex |  |  |  |  |  |  | 0.21 |  |  | 0.11 |
| Male | 30 | 239 ±514 | 35 | 328 ±488 | 35 | 188 ± 461 |  |  | -19 (-230 to 193) |  |
| Female | 45 | 287 ±509 | 30 | 92 ±257 | 41 | 81 ± 494 |  |  | 201 (31 to 371) |  |
| Race |  |  |  |  |  |  | 0.32 |  |  | 0.17 |
| White | 58 | 294 ±547 | 50 | 180 ±391 | 57 | 113 ± 530 |  |  | 150 (-11 to 310) |  |
| Non-White | 17 | 180 ±341 | 15 | 351 ±469 | 18 | 163 ± 271 |  |  | -69 (-289 to 152) |  |
| Relationship status |  |  |  |  |  |  | 0.71 |  |  | 0.65 |
| Married | 41 | 284 ±546 | 34 | 293 ±428 | 37 | 152 ± 545 |  |  | 65 (-135 to 264) |  |
| Not married | 34 | 248 ±466 | 31 | 138 ±386 | 39 | 110 ± 413 |  |  | 126 (-49 to 300) |  |
| Household income |  |  |  |  |  |  | 0.74 |  |  | 0.91 |
| ≥$40,000/y | 35 | 256 ±528 | 31 | 236 ±433 | 36 | 96 ± 495 |  |  | 96 (-107 to 298) |  |
| <40,000/y | 38 | 262 ±480 | 31 | 190 ±404 | 37 | 175 ± 485 |  |  | 81 (-103 to 265) |  |
| BMI |  |  |  |  |  |  | 0.62 |  |  | 0.41 |
| ≤30 | 42 | 340 ±592 | 39 | 224 ±382 | 34 | 165 ± 541 |  |  | 144 (-52 to 341) |  |
| >30 | 33 | 175 ±364 | 26 | 212 ±463 | 42 | 103 ± 426 |  |  | 30.82 (-145 to 207) |  |
| Smoking status |  |  |  |  |  |  | 0.59 |  |  | 0.69 |
| Never smoker | 35 | 275 ±494 | 27 | 151 ±412 | 30 | 129 ± 432 |  |  | 135 (-57 to 327) |  |
| Former or current smoker | 40 | 262 ±526 | 37 | 275 ±415 | 43 | 101 ± 486 |  |  | 80 (-105 to 265) |  |
| Duration of back symptoms |  |  |  |  |  |  | 0.39 |  |  | 0.17 |
| ≤6 months | 6 | 144 ±277 | 10 | 331 ±513 | 6 | 353 ± 284 |  |  | -195 (-592 to 202) |  |
| >6 months | 69 | 279 ±523 | 55 | 199 ±394 | 70 | 111 ± 488 |  |  | 129 (-13 to 270) |  |
| Duration of leg symptoms |  |  |  |  |  |  | 0.63 |  |  | 0.45 |
| ≤6 months | 18 | 280 ±444 | 21 | 266 ±498 | 19 | 268 ± 505 |  |  | 13.14 (-260 to 286) |  |
| >6 months | 57 | 264 ±530 | 44 | 197 ±370 | 57 | 85 ± 465 |  |  | 130.4 (-23 to 283) |  |
| Diagnostic imaging results |  |  |  |  |  |  | 0.75 |  |  | 0.79 |
| Central canal stenosis only | 6 | 151 ±300 | 5 | 296 ±252 | 6 | 141 ± 433 |  |  | -61 (-426 to 304) |  |
| Lateral canal stenosis only | 30 | 263 ±636 | 20 | 146 ±248 | 38 | 176 ± 406 |  |  | 97 (-156 to 351) |  |
| Central/lateral canal stenosis | 36 | 272 ±421 | 36 | 239 ±474 | 31 | 63 ± 573 |  |  | 114 (-87 to 316) |  |
| Hip osteoarthritis |  |  |  |  |  |  | 0.03 |  |  | 0.01 |
| Yes | 13 | 608 ±693 | 10 | 133 ±384 | 12 | 87 ± 666 |  |  | 500 (71 to 929) |  |
| No | 62 | 197 ±434 | 55 | 235 ±420 | 64 | 139 ± 441 |  |  | 13 (-120 to 147) |  |
| Knee osteoarthritis |  |  |  |  |  |  | 0.72 |  |  | 0.49 |
| Yes | 24 | 133 ±340 | 16 | 176 ±236 | 28 | 61 ± 454 |  |  | 30 (-159 to 219) |  |
| No | 51 | 331 ±562 | 49 | 233 ±458 | 48 | 171 ± 493 |  |  | 129 (-44 to 302) |  |
| No. of comorbidities |  |  |  |  |  |  | 0.92 |  |  | 0.86 |
| ≤4 | 38 | 236 ±517 | 43 | 187 ±374 | 36 | 114 ±495 |  |  | 82 (-98 to 263) |  |
| >4 | 37 | 301 ±504 | 22 | 283 ±484 | 40 | 145 ±470 |  |  | 107 (-94 to 307) |  |
| Ankle-brachial index |  |  |  |  |  |  | 0.98 |  |  | 0.91 |
| <1 | 29 | 308 ±506 | 25 | 250 ±420 | 23 | 157 ±395 |  |  | 103 (-106 to 312) |  |
| ≥1 | 46 | 242 ±513 | 40 | 200 ±413 | 53 | 119 ±514 |  |  | 88 (-85 to 262) |  |
| SSSQ total score |  |  |  |  |  |  | 0.53 |  |  | 0.41 |
| < 31 | 40 | 336 ±647 | 30 | 276 ±499 | 39 | 121 ±502 |  |  | 148 (-73 to 369) |  |
| > 31 | 35 | 189 262 | 35 | 170 ±321 | 37 | 141 ±459 |  |  | 34 (-93 to 162) |  |
| Walking distance on SPWT |  |  |  |  |  |  | 0.53 |  |  | 0.91 |
| < 280 | 35 | 237 ±374 | 34 | 140 ±325 | 42 | 136 ±231 |  |  | 99 (-43 to 241) |  |
| > 280 | 40 | 295 ±605 | 31 | 307 ±482 | 34 | 123 ±674 |  |  | 84 (-154 to 322) |  |
| Oswestry Disability Index |  |  |  |  |  |  | 0.79 |  |  | 0.56 |
| ≤40 | 47 | 335 ±576 | 37 | 276 ±457 | 44 | 162 ±504 |  |  | 121 (-67 to 310) |  |
| >40 | 28 | 155 ±347 | 28 | 144 ±341 | 32 | 87 ±445 |  |  | 40 (-133 to 215) |  |
| Leg pain intensity |  |  |  |  |  |  | 0.95 |  |  | 0.90 |
| ≤6 | 48 | 274 ±548 | 39 | 228 ±426 | 44 | 121 ±509 |  |  | 103 (-77 to 282) |  |
| >6 | 27 | 257 ±437 | 26 | 206 ±401 | 32 | 144 ±441 |  |  | 85 (-112 to 283) |  |
| Back pain intensity |  |  |  |  |  |  | 0.96 |  |  | 0.99 |
| ≤6 | 31 | 295 ±659 | 35 | 246 ±441 | 28 | 138 ±543 |  |  | 97 (-172 to 365) |  |
| >6 | 44 | 249 ±374 | 30 | 188 ±383 | 48 | 126 ±443 |  |  | 99 (-52 to 250) |  |
| Gait speed |  |  |  |  |  |  | 0.80 |  |  | 0.56 |
| <1 m/s | 47 | 217 ±459 | 38 | 209 ±386 | 50 | 107 ±422 |  |  | 66 (-86 to 219) |  |
| ≥1 m/s | 28 | 353 ±579 | 27 | 234 ±456 | 26 | 175 ±578 |  |  | 147 (-103 to 397) |  |
| Physical activity |  |  |  |  |  |  | 0.99 |  |  | 0.96 |
| <165 min/d | 40 | 208 ±410 | 38 | 150 ±409 | 43 | 86 ±470 |  |  | 92 (-73 to 257) |  |
| ≥165 min/d | 34 | 347 ±605 | 24 | 311 ±421 | 31 | 224 ±472 |  |  | 84 (-157 to 325) |  |
| PROMIS Depression |  |  |  |  |  |  | 0.89 |  |  | 0.73 |
| <55 | 61 | 274 ±537 | 50 | 233 ±424 | 61 | 131 ±513 |  |  | 97 (-60 to 254) |  |
| >55 | 12 | 311 ±322 | 15 | 173 ±383 | 15 | 130 ±313 |  |  | 159 (-74 to 393) |  |
| Tampa Scale of Kinesiophobia |  |  |  |  |  |  | 0.77 |  |  | 0.50 |
| <26 | 39 | 316 ±584 | 32 | 223 ±406 | 32 | 131 ±473 |  |  | 139 (-77 to 356) |  |
| >26 | 36 | 215 ±411 | 33 | 216 ±426 | 44 | 130 ±488 |  |  | 48 (-130 to 227) |  |
| Treatment expectancy of MTE |  |  |  |  |  |  | 0.28 |  |  | 0.14 |
| ≤42 | 29 | 174 ±520 | 28 | 214 ±357 | 29 | 191 ±365 |  |  | -28 (-245 to 189) |  |
| >42 | 46 | 327 ±496 | 37 | 223 ±456 | 47 | 93 ±538 |  |  | 177 (-6 to 359) |  |
| * No defined range; total distance in meters walked in 0 to 30 minutes. Less walking capacity indicates worse physical function.  † Test for interaction (i.e. statistical effect modification) between baseline characteristic and treatment | | | | | | | | |  |  |
